# DCA1 Acts as a Transcriptional Co-activator of DST and Contributes to Drought and Salt Tolerance in Rice

**DOI:** 10.1371/journal.pgen.1005617

**Published:** 2015-10-23

**Authors:** Long-Gang Cui, Jun-Xiang Shan, Min Shi, Ji-Ping Gao, Hong-Xuan Lin

**Affiliations:** National Key Laboratory of Plant Molecular Genetics, CAS Centre for Excellence in Molecular Plant Sciences and Collaborative Innovation Center of Genetics and Development, Shanghai Institute of Plant Physiology and Ecology, Shanghai Institutes for Biological Sciences, Chinese Academy of Sciences, Shanghai, China; The University of North Carolina at Chapel Hill, UNITED STATES

## Abstract

Natural disasters, including drought and salt stress, seriously threaten food security. In previous work we cloned a key zinc finger transcription factor gene, *D*rought and *S*alt *T*olerance (*DST*), a negative regulator of drought and salt tolerance that controls stomatal aperture in rice. However, the exact mechanism by which DST regulates the expression of target genes remains unknown. In the present study, we demonstrated that DST Co-activator 1 (DCA1), a previously unknown CHY zinc finger protein, acts as an interacting co-activator of DST. DST was found to physically interact with itself and to form a heterologous tetramer with DCA1. This transcriptional complex appears to regulate the expression of *peroxidase 24 precursor* (*Prx 24*), a gene encoding an H_2_O_2_ scavenger that is more highly expressed in guard cells. Downregulation of *DCA1* significantly enhanced drought and salt tolerance in rice, and overexpression of *DCA1* increased sensitivity to stress treatment. These phenotypes were mainly influenced by DCA1 and negatively regulated stomatal closure through the direct modulation of genes associated with H_2_O_2_ homeostasis. Our findings establish a framework for plant drought and salt stress tolerance through the *DCA1-DST-Prx24* pathway. Moreover, due to the evolutionary and functional conservation of *DCA1* and *DST* in plants, engineering of this pathway has the potential to improve tolerance to abiotic stress in other important crop species.

## Author Summary

Drought and salt are two of the most serious threats to food production worldwide, and research on stress tolerance in crops is important for future food security. In this study we identified DCA1, a transcriptional co-activator of DST that is conserved in the world’s three major crops. DCA1 participates in stress tolerance by controlling stomatal aperture through modulation of H_2_O_2_ homeostasis in guard cells. This finding not only increases our understanding of the molecular mechanism by which plants withstand harsh environmental conditions, but it may also facilitate future molecular breeding and genetic engineering of drought- and salt-tolerant crops.

## Introduction

How to feed a growing population that is expected to reach roughly 9 billion by the middle of this century is among the major challenges of our time [1]. Modern agriculture has greatly improved food production [2], but progress towards avoiding the negative effects of climate change and diminishing soil conditions has been insufficient. Most worryingly, many of the plants upon which we depend for food production are particularly sensitive to environmental stress [3]. Droughts are likely to be more frequent as global warming accelerates, and rising sea levels will result in the loss of productive agricultural land to water infiltration and increased soil salinity. Together these unfavorable factors pose a huge threat to food security, and studying drought and salt tolerance in crops is becoming increasingly urgent.

Many previous studies on drought and salt tolerance in plants have mainly focused on the model species *Arabidopsis thaliana*. Years of effort have revealed that the responses of plants to water stress are controlled by complex regulatory signalling events mediated by Ca^2+^ [4, 5], abscisic acid (ABA) [6–8], reactive oxygen species (ROS) [9, 10], ion transport [4, 11–13], and the activities of transcription factors (TFs) [14]. Stomata are often described as the guardians of leaves which are of course the photosynthetic organs in which food manufacturing occurs. The main function of stomata, which are formed from pairs of guard cells, is to allow gases to move rapidly into and out of the leaf. However, this evolved trait poses a problem for plants since they face the predicament of taking up CO_2_ through stomata while attempting to minimize water loss through these pores. The ability to effectively control the balance between photosynthesis and transpiration in accordance with the external environment is an impressive evolutionary achievement.

ROS such as H_2_O_2_ are a product of the incomplete reduction of molecular oxygen, and ROS production is widely considered a symptom of cellular dysfunction. ROS participate in cell death and may also function as signalling molecules. Increasing evidence suggests that ROS function as second messengers in stomatal aperture control [9, 15]. In 1996, McAinsh *et al*. first reported that ROS induced stomatal closure and inhibited stomatal opening by increasing the concentration of cytosolic free calcium ([Ca^2+^]cyt) in *Commelina communis* [16]. A detailed follow-up study reported that ABA-stimulated ROS accumulation induced stomatal closure via the activation of plasma membrane calcium channels in Arabidopsis [9].

Since plants are sessile organisms, they must have highly evolved sophisticated mechanisms to detect and respond to environmental perturbations. Changes in the expression of stress-related genes are an important part of the plant response to environmental stress. Numerous transcription factors including APETALA 2/ethylene-responsive element binding factor (AP2/ERF), dehydration responsive element binding protein (DREB)/C-repeat-binding factor (CBF), ABA-responsive element binding protein (AREB)/ABA-responsive element-binding factor (ABF), No apical meristem, Arabidopsis transcription activation factor and Cup-shaped cotyledon (NAC) are associated with plant abiotic stress responses [14]. Regulation of target genes by TFs is a highly complex and delicately balanced process. In most cases TFs do not function alone but recruit partner proteins (cofactors) to form transcription initiation complexes [17]. Cofactors are transcription factor interacting proteins that either activate or repress the transcription of target genes, and numerous examples have been reported in animals including humans, but few have been identified in plants. Arabidopsis HAIRY MERISTEM (HAM) family proteins were recently found to act as conserved interacting cofactors with the transcription factor WUSCHEL (WUS) to drive downstream transcriptional programs that help promote shoot stem cell proliferation [18]. Another study demonstrated that HYPOXIA RESPONSE ATTENUATOR1 (HRA1) interacts with ethylene-responsive factor group VII transcription factor (ERF-VII TF) RAP2.12 to negatively modulate its activity under hypoxia [19].

Efforts have also been made to determine the underlying physiological, genetic and molecular mechanisms mediating drought and salt tolerance in crops such as rice. *Stress-responsive NAC 1* (*SNAC1*) is a member of the rice NAC TF family that is specifically induced in guard cells in drought conditions, and overexpression of *SNAC1* in rice significantly enhanced drought tolerance [20]. We previously isolated the C_2_H_2_ zinc finger transcription factor DST that negatively regulates stress tolerance in rice [21]. DST regulates stomatal aperture by modulating the expression of genes related to ROS homeostasis. However, these studies are fragmentary. The exact mechanisms by which these TFs regulate the expression of target genes remain unknown. In the present study, we identified the CHY zinc finger protein DCA1, an interacting partner of DST. Homologs of DCA1 in rice and Arabidopsis were recently shown to increase stomatal opening and were upregulated by heat stress [22]. However the exact molecular function of this protein, the pathways involved and the phenotypes of plants in which DCA1 is modified remain unknown. In this research, we revealed that DCA1 forms a heterologous tetramer with DST and positively regulates DST activity. This transcriptional complex regulates the expression of *Prx24*, an H_2_O_2_ scavenger preferentially expressed in guard cells. Together, the DCA1-DST-Prx24 pathway contributes to stomatal movement via regulating ROS homeostasis under stress conditions. These findings may facilitate the engineering of crops with improved drought and salt tolerance.

## Results

### Physical Interaction between DCA1 and DST

**Fig 1:**
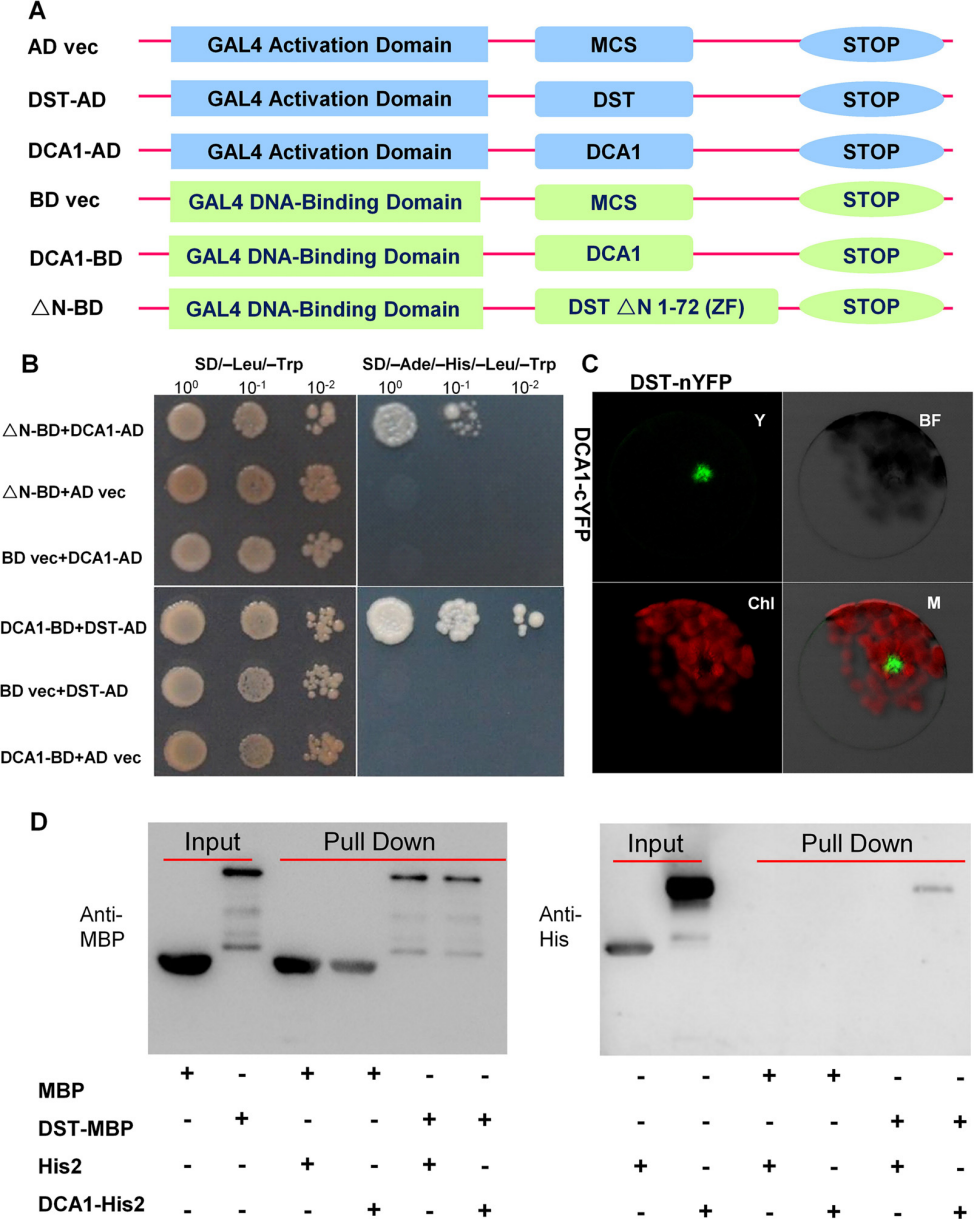
Physical interaction between DCA1 and DST. (A) Yeast two-hybrid constructs using native and variant DST and DCA1 fragments. (B) Growth of yeast cells harboring various combinations of constructs. SD/-Leu/-Trp and SD/-Ade/-His/-Leu/-Trp were used as nonselective and selective media, respectively. The assay revealed an interaction between DCA1 and both full-length DST and the C-terminal fragment of DST (△N 1–72 DST). (C) BiFC assay in Arabidopsis protoplasts revealing the interaction between DST and DCA1 *in vivo*. Y, YFP; BF, Bright Field; Chl, Chlorophyll; M, Merge. (D) *In vitro* pull-down of His2-tagged DCA1 using MBP-tagged DST as determined by immunoblotting with anti-MBP antibody (left) and anti-His antibody (right).

Most TFs do not function alone. Rather, they recruit intermediary proteins (cofactors) to initiate transcription effectively. In humans, several hundred putative co-regulators have been identified [17]. To identify cofactors of DST, we performed yeast two-hybrid screening with a ZH11 (*Oryza sativa* L. *japonica*. cv. ZhongHua 11) leaf cDNA library. A version of DST with the self-activation domain removed (amino acids 1–72 removed from the N-terminus, including the zinc finger domain, ΔN) was used as bait. Dozens of potential targeting proteins were found including a U-box domain containing protein, Elongation Factor 1-alpha, a protein similar to Calcium-dependent protein kinase-related kinase, and the CHY zinc finger protein DCA1. To further verify the interaction between these proteins, we used DCA1 and DST as bait and prey, respectively (Fig 1A), and their interaction was confirmed through additional yeast two-hybrid assays. DCA1 was able to interact with DST independently of its C_2_H_2_ zinc finger domain which likely functions in DNA binding (Fig 1B). The DST-DCA1 interaction was also confirmed via bimolecular fluorescence complementation (BiFC) assays in Arabidopsis protoplasts *in vivo*, in which DST was fused to the amino-termini half of yellow fluorescent protein (YFP) (DST-nYFP) and DCA1 was fused to the carboxy-termini half of YFP (DCA1-cYFP). The YFP signal was mainly restricted to nuclei when DST-nYFP was cotransformed with DCA1-cYFP (Fig 1C). Consistent with these results, an *in vitro* pull-down assay using recombinant DST fused to maltose binding protein (DST-MBP) and His2-tagged DCA1 (DCA1-His2) that were expressed in *E*. *coli* showed that DST-MBP but not MBP was able to pull down DCA1-His2 (Fig 1D). These results confirm the direct interaction between DCA1 and DST both *in vitro* and *in vivo*.

### *DCA1* Characterization

**Fig 2:**
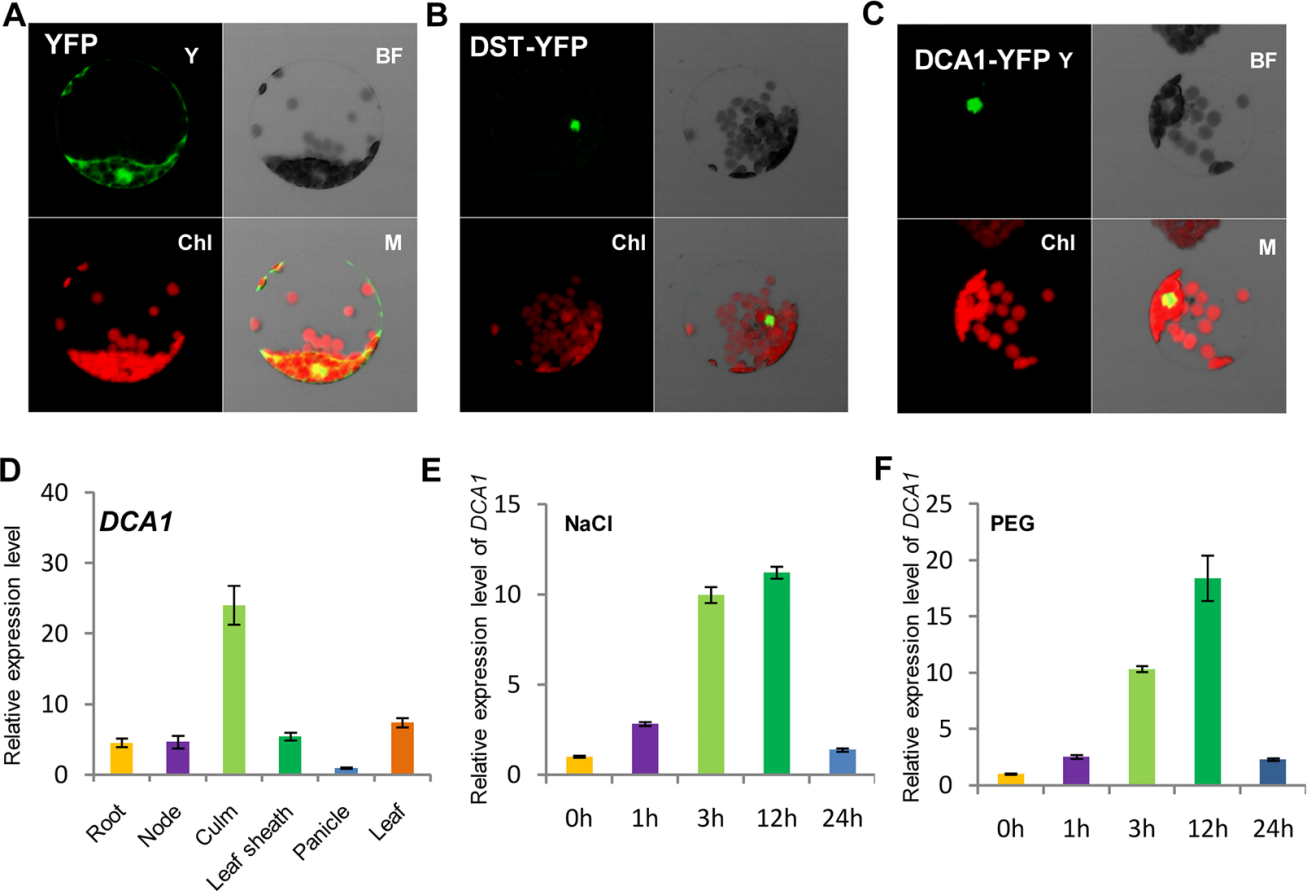
Subcellular localization and tissue-specific expression of DCA1. Subcellular localization of (A) YFP, (B) DST-YFP and (C) DCA1-YFP in Arabidopsis protoplasts. Both DST and DCA1 are located predominantly in the nucleus. Y, YFP; BF, Bright Field; Chl, Chlorophyll; M, Merge. (D) Expression pattern of *DCA1* in different tissues of ZH11. Data represent means ± sd (n = 3). (E, F) Relative expression levels of *DCA1* in the leaves of plants treated with NaCl (E) or PEG (F). Data are means ± sd (n = 3).

Careful analysis of the structure of DCA1 identified a CHY zinc finger domain and a C_3_H_2_C_3_ or RING-H2 domain (S1A Fig). A database search for homologous proteins identified conserved CHY zinc finger domains in various different species, but the C_3_H_2_C_3_ domain appeared not to be conserved (S1B Fig). To characterize *DCA1*, we investigated the subcellular localization of DST-YFP and DCA1-YFP fusion proteins in Arabidopsis protoplasts. YFP fluorescence was apparent in both the cytoplasm and nuclei of control protoplasts transformed with the YFP-vector. In contrast YFP fluorescence was mainly restricted to nuclei in DST-YFP and DCA1-YFP transformants (Fig 2A–2C). Investigation of the tissue-specific expression of *DCA1* by qRT-PCR revealed a similar expression pattern to that of *DST*, with both genes expressed at relatively high levels in leaves and culms (Fig 2D and S2A Fig). We also tested whether the expression of *DCA1* was regulated by drought and salt stress using qRT-PCR and found that *DCA1* is upregulated rapidly after 1–3 h of drought or salt treatment, with expression peaking at 12 h and normal expression resumed 24 h after treatment (Fig 2E and 2F).

### Overexpression of *DCA1* Reduces Stress Tolerance Ability Consisting with DST

**Fig 3:**
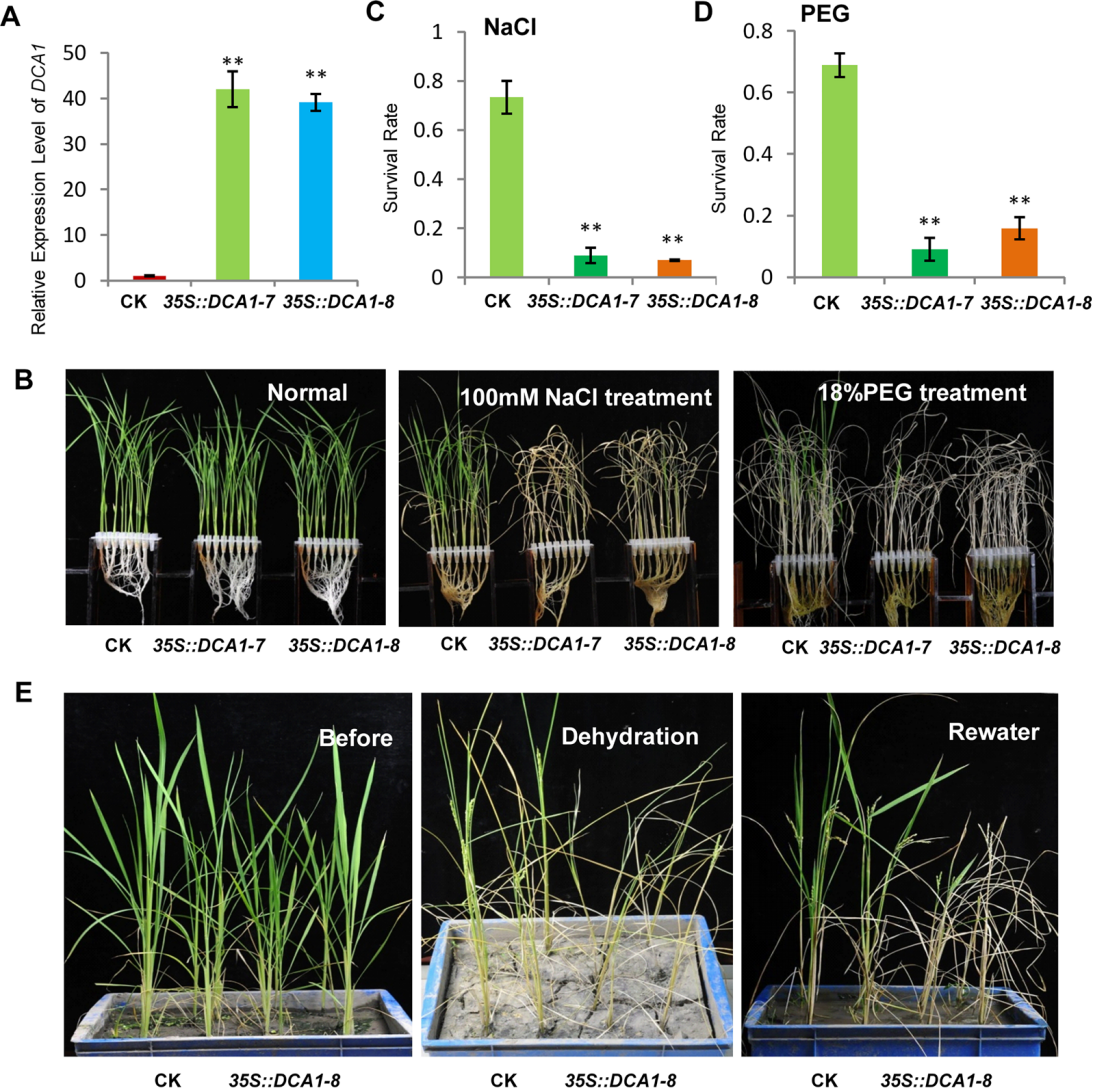
Overexpression of *DCA1* reduces stress tolerance in rice. (A) Expression levels of *DCA1* in vector-only ZH11 (CK), *35S*::*DCA1-7* (*DCA1-7*) and *35S*::*DCA1-8* (*DCA1-8*) plants were measured and normalized against *Actin* (data represent means ± sd, n = 3). (B) CK, *35S*::*DCA1-7* and *35S*::*DCA1-8* plants grown under normal conditions for 12 days (left), 12-day-old seedlings transferred to 100 mM NaCl for 10 days and recovered for 7 days (middle), or transferred to 18% PEG for 13 days and recovered for 7 days (right). (C) Relative survival rates after NaCl treatment (n = 3 biological replicates, 24 plants of each replicate). Data represent means ± sd (n = 3). (D) Relative survival rates after PEG treatment (n = 3 biological replicates, 24 plants of each replicate). Data represent means ± sd (n = 3). (E) Plants grown in soil under normal conditions for 63 days (left), dehydrated for 13 days (middle) and recovered for 16 days (right). Significant differences were determined using the Student’s t-test (**P <0.01).

**Fig 4:**
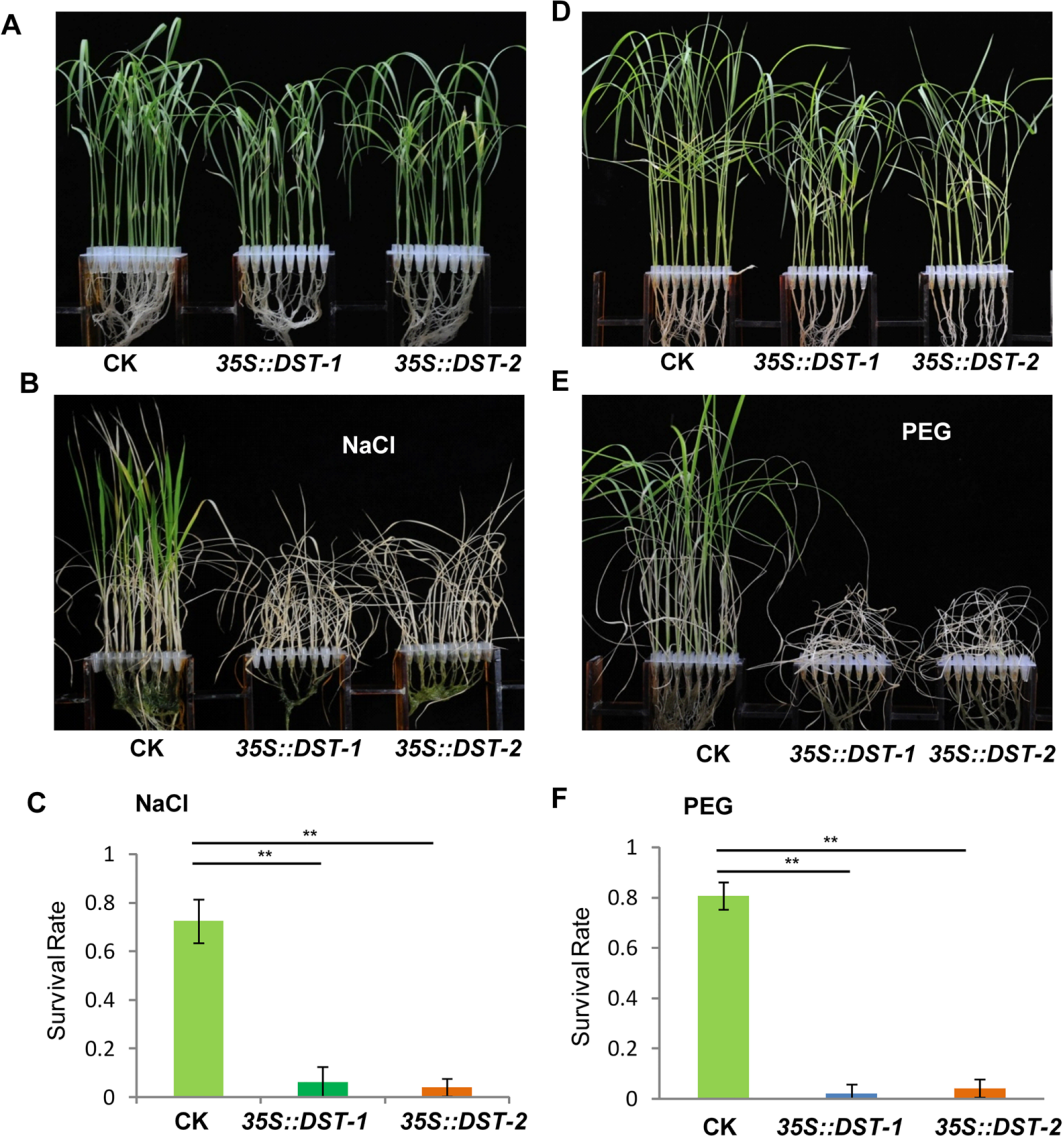
*DST*-overexpressing like *35S*::*DCA1* plants exhibit reduced drought and salt tolerance. (A) Control (CK; vector-only ZH11), *35S*::*DST-1* and *35S*::*DST-2* plants grown under normal conditions for 12 days. (B) 12-day-old seedlings treated with 100 mM NaCl for 8 days and recovered for 7 days. (C) Survival percentage of plants following NaCl treatment (n = 3 biological replicates, 24 plants of each replicate). Data represent means ± sd (n = 3). (D) Plants grown under normal conditions for 12 days. (E) 12-day-old seedlings treated with 18% PEG for 10 days and recovered for 7 days. (F) Survival percentage of plants after PEG treatment (n = 3 biological replicates, 24 plants of each replicate). Data represent means ± sd (n = 3). Significant differences were determined using the Student’s t-test (**P <0.01).

To dissect the physiological functions of *DCA1* in plants, we generated transgenic rice (ZH11) overexpressing *DCA1* under the control of the enhanced cauliflower mosaic virus 35S promoter. qRT-PCR assays confirmed that *DCA1* expression was enhanced in all eight overexpression lines, compared with vector-only control ZH11 plants (CK). Two transgenic lines, *35S*::*DCA1-7* and *35S*::*DCA1-8*, were selected for further analysis (Fig 3A), and the tolerance to drought and salt stress was investigated. Twelve-day-old CK, *35S*::*DCA1-7* and *35S*::*DCA1-8* seedlings grown in normal conditions were treated with 100 mM NaCl for 10 days or with 18% PEG for 13 days. As shown in Fig 3B–3D, almost all of the *35S*::*DCA1* seedlings died, while approximately 70% of the vector-only control plants survived, following a subsequent 7 day recovery in normal conditions. The relative chlorophyll content decreased to ~13% in *35S*::*DCA1-8* plants vs. ~27% in vector-only control plants after drought treatment (S3A Fig). Fresh weight similarly decreased to ~52% in *35S*::*DCA1-8* plants vs. ~80% in vector-only control plants after drought treatment (S3C Fig). These results indicated that *35S*::*DCA1* plants were extremely sensitive to drought and salt stress compared with CK plants. Since there are many differences between laboratory and field stress conditions, we examined the responses of the *35S*::*DCA1* overexpression plants to drought stress in soil and obtained similar results. Specifically, *35S*::*DCA1* plants were much more sensitive to drought stress than CK plants (Fig 3E). In a previous study, we failed to obtain plants overexpressing *DST*, and their phenotypes under stress conditions remain unknown [21]. However after many years of effort, various *35S*::*DST* lines have been prepared and qRT-PCR analysis revealed that expression of *DST* was only increased 1–2-fold in these plants (S5A Fig). This may be because strong overexpression of this gene causes death. 12-day-old CK, *35S*::*DST-1* and *35S*::*DST-2* seedlings grown under normal conditions were stressed with either 100 mM NaCl or 18% PEG and allowed to recover for 7 days. Almost all of the *35S*::*DST* seedlings died, while approximately 70% (NaCl) and 80% (PEG) of the vector-only control plants survived (Fig 4). The relative chlorophyll content decreased to ~7% in *35S*::*DST-1* plants vs. ~27% in vector-only control plants (S3A Fig). Similarly, fresh weight decreased to ~53% in *35S*::*DST-1* plants vs. ~80% in vector control plants (S3C Fig). These results showed that *35S*::*DST* plants were much more sensitive to drought and salt stress than controls, consistent with the results obtained for *35S*::*DCA1* (Fig 3). We also checked whether the reduced stress tolerance of *35S*::*DCA1* plants was caused by changes in *DST* expression, but the results suggested this was not the case (S5B Fig). We concluded that DCA1 may therefore function in cooperation with DST in the stress response.

### The *DCA1* Knock-Down Plants Exhibits Improved Stress Tolerance

**Fig 5:**
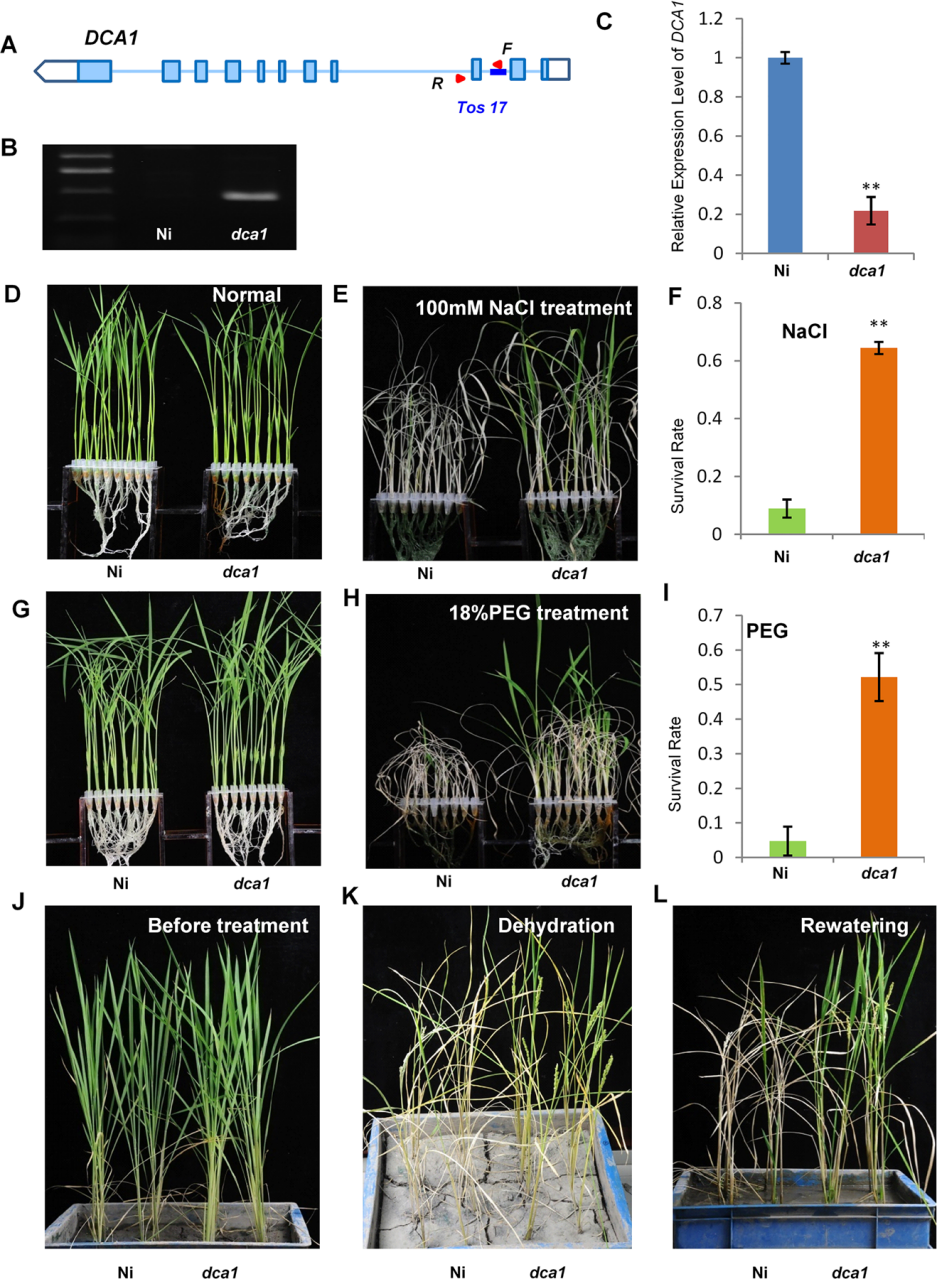
The *DCA1* knockdown mutant has improved stress tolerance. (A, B) Testing of the DCA1 knockdown mutant showing *Tos17* insertion in the second intron of *DCA1*. Red triangles represent primers used for mutant examination. F primer was on the *Tos17* fragment and R was on genome. (C) *DCA1* expression in the *dca1* mutant. Data represent means ± sd (n = 3). (D, E) Nipponbare (Ni) and *dca1* seedlings grown under normal conditions for 12 days (D), transferred to 100 mM NaCl for 9 days and recovered for 7 days (E). (F) Relative survival rates of Ni and *dca1* after NaCl treatment (n = 3 biological replicates, 24 plants of each replicate). Data represent means ± sd (n = 3). (G, H) Ni and *dca1* seedlings grown under normal conditions for 12 days (G), transferred to 18% PEG for 10 days and recovered for 7 days (H). (I) Relative survival rates of Ni and *dca1* after PEG treatment (n = 3 biological replicates, 24 plants of each replicate). Data represent means ± sd (n = 3). (J) Ni and *dca1* grown in soil under normal conditions for 66 days, (K) dehydrated for 14 days and (L) recovered for 16 days. Significant differences were determined using the Student’s t-test (**P <0.01).

A *Tos17* mutant (NF7038) of *DCA1* was obtained from the Rice Genome Resource Center. The *Tos17* fragment was inserted into the second intron of *DCA1*, and this reduced *DCA1* expression levels by ~80% compared with wild-type *Oryza sativa* L. *japonica*. cv. Nipponbare. These results indicate that this mutant (designated *dca1*) was a genuine *DCA1* knockdown mutant (Fig 5A–5C).

We next evaluated whether *dca1* plants possessed enhanced tolerance to drought or salt stress. 12-day-old seedlings of Ni (Nipponbare) and *dca1* grown under normal conditions (Fig 5D and 5G) were treated with 100 mM NaCl for 9 days (Fig 5E) or 18% PEG for 10 days (Fig 5H) and recovered for 7 days. Approximately 10% of Ni plants survived following a subsequent 7 day recovery period after salt stress, compared with 62% of *dca1* plants (Fig 5F). Similarly, the survival rate following recovery from drought stress was significantly increased in the *dca1* mutant compared with Ni plants (Fig 5I). The relative chlorophyll content decreased to ~5% in Ni vs. ~16% in *dca1* plants following drought treatment (S3B Fig), and the fresh weight decreased to ~43% and ~89% in Ni and *dca1* plants, respectively (S3D Fig). These results indicated that *dca1* plants were more tolerant to abiotic stress than Ni. Since *dca1* is a *Tos17* insertion mutant, we knew there may be several copies of the insertion, and Southern blotting revealed more than 10 copies (S4A Fig). We therefore designed an artificial mircoRNA to elicit knockdown of *DCA1*, and expression of *DCA1* in the silenced variant was reduced by 50–80% compared with CK plants. *DCA1* microRNA transgenic plants were also more tolerant of drought and salt stress than CK plants (S4B, S4C, S4E and S4F Fig). Dehydration treatment of 66-day-old Ni and *dca1* plants grown in soil under normal conditions for 14 days and rewatered for 16 days showed that *dca1* was more tolerant to soil dehydration than Ni (Fig 5J–5L). Moreover, *dca1* seeds could be harvested from the surviving tillers after re-irrigation (S5C and S5D Fig). Together these results indicated that *DCA1* knockdown plants exhibited improved tolerance to drought and salt stress, consistent with the phenotype of the *DST* mutant *dst* [21].

### DCA1 Acts as a Co-activator of DST and Positively Regulates the Expression of Genes Downstream of DST

**Fig 6:**
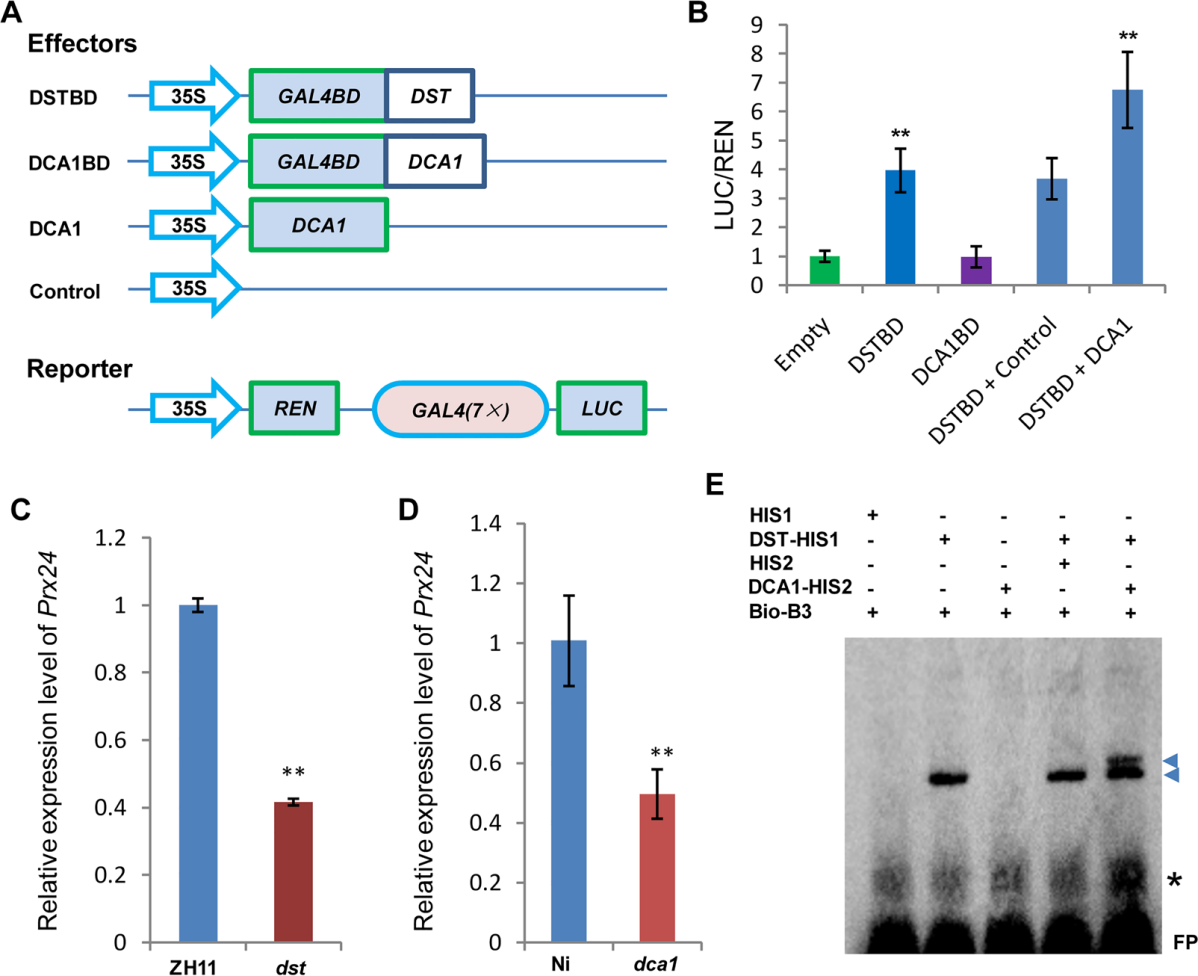
DCA1 acts as an interaction co-activator of DST. (A) Schematic diagram of constructs used in Dual Luciferase assays. (B) Ratio of firefly luciferase (LUC) to Renilla luciferase (REN) activity in Arabidopsis protoplasts cotransformed with different reporter constructs and effectors shown in (A). The results show that DST acts as a transcriptional activator, while DCA1 enhances the transcriptional activation function of DST. Data represent means ± sd (n = 3). (C) Real-time PCR quantification of the expression of the DST downstream target gene *Prx24* in ZH11 and *dst*. Data represent means ± sd (n = 3). (D) Real-time PCR quantification of the expression of *Prx24* in Ni and *dca1*. Data represent means ± sd (n = 3). (E) EMSA of DST protein with DCA1 protein on biotin-tagged B3 DNA containing the DST binding site sequence. Triangles and asterisks indicate shifted bands and nonspecific binding, respectively. FP, free probe. Significant differences were determined using the Student’s t-test (**P <0.01).

We previously demonstrated that DST is a transcription factor with transactivational activity [21]. However, we found that DCA1 does not exhibit this activity directly, since yeast harboring the DCA1-BD construct did not grow on SD/-Ade-His-Leu-Trp medium (Fig 1B). We therefore conducted dual luciferase assays in Arabidopsis protoplasts to confirm the direct effects of the DCA1-DST interaction on gene expression. Seven copies of the GAL4 binding sequence are located before the translational start site of the REN reporter and act as putative *cis*-acting elements. Compared with empty-vector controls, the REN/LUC ratio was moderately upregulated by DST alone but markedly upregulated when DCA1 and DST were both present, indicating a positive role for this interaction in regulating DST transcriptional activity (i.e., DCA1 enhances the transcriptional activity of DST; Fig 6A and 6B). *Peroxidase 24 precursor* (*Prx 24*) is an H_2_O_2_ scavenger and target gene of DST, as verified by ChIP and electrophoretic mobility shift assay (EMSA) [21]. Our qRT-PCR results showed that *Prx24* was downregulated in *dst*, *dca1* and *DmiR* (*DCA1* artificial microRNA) leaves (Fig 6C and 6D and S4D Fig) and upregulated in *35S*::*DST* and *35S*::*DCA1* leaves (S6A and S6B Fig).

Although we know that *Prx24* is a downstream gene of this transcriptional regulation module, phenotypes of transgenic plants under stress conditions remain unknown. So we generated overexpression plants of this gene in ZH11. *Prx24*-overexpressing plants became sensitive to drought and salt stress to an extent comparable to *DCA1* and *DST*-overexpressing plants in a ZH11 background (S6C Fig). We identified the DST binding sequence (DBS), 5’-TGCTANNATTG-3’, using a bacterial one-hybrid system [21], and EMSA results showed that His1-DST but not the His1 tag alone could bind to B3, a DNA fragment containing this conserved site (DBS) (Fig 6E). We next wanted to determine if DCA1 could also bind to the DBS, and further EMSA results revealed that His2-DCA1 and His1-DST could bind to the DBS, while His2-DCA1 alone could not (Fig 6E). It is possible that the B3 sequence that we used did not include a DCA1 binding site, or perhaps DCA1 was not associated with DNA at all. To verify our hypothesis, we used the longer promoter sequence of *Prx24* described in our previous research [21], and EMSA results were similar and confirmed that DST but not DCA1 could bind to the fragment (S6D Fig). Together, our combined genetic, physiological and molecular evidence indicated that DCA1 functions as an interaction co-activator in DST-mediated transcriptional programs.

### DCA1 Positively Regulates Stomatal Aperture

**Fig 7:**
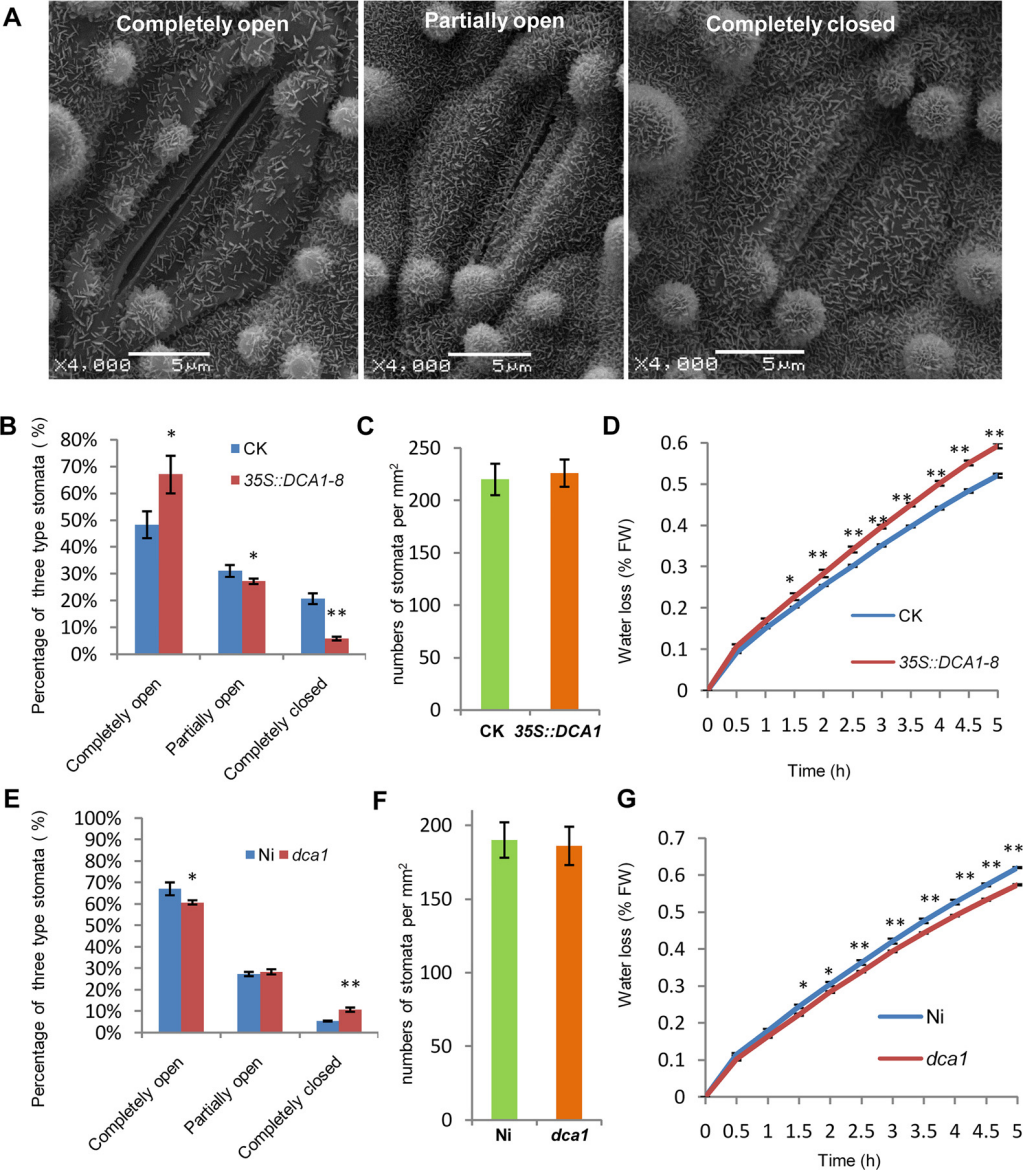
*DCA1* affects stomatal aperture. (A) Cryo scanning electron microscopy (Cryo SEM) images of three levels of stomatal opening of ZH11. (B) The percentage of the three levels in CK and *35S*::*DCA1-8* plants under normal conditions (n = 8 samples, ~10 stomata per sample; 97 stomata for CK; 103 stomata for *35S*::*DCA1-8*). (C) Stomatal density of the middle leaves of CK and *35S*::*DCA1-8* (data represent means ± sd, n = 10). Three random scopes were used for each repeat (253.3 μm × 253.3 μm for each scope). (D) Water loss in CK and *35S*::*DCA1-8* leaves (n = 3 biological replicates, each containing 10 fully expanded leaves from 25-day-old plants). (E) The percentage of the three levels in Ni and *dca1* plants (n = 8 samples, ~10 stomata per sample; 73 stomata for Ni; 102 stomata for *dca1*). (F) Stomatal density of the middle leaves of Ni and *dca1* (data represent means ± sd, n = 10). Three random scopes were used in each repeat (422.2 μm × 422.2 μm for each scope). (G) Water loss in Ni and the *dca1* mutant (n = 3 biological replicates, each containing 10 fully expanded leaves from 25-day-old plants). Data represent means ± sd. Significant differences were determined using the Student’s t-test (*P <0.05, **P <0.01).

Since DST negatively regulates stomatal closure to improve drought and salt tolerance [21], we investigated whether stomatal aperture was also altered in *35S*::*DCA1* and *dca1* plants using Cryo scanning electron microscopy (Cryo SEM). Three levels of stomatal opening in different plants were observed (Fig 7A and S7A Fig) and statistical analysis revealed that 6% of stomata were completely closed in *35S*::*DCA1* plants vs. 21% in CK plants, while 67% were completely open in *35S*::*DCA1* plants vs. only 48% in CK plants. The percentage of partially open stomata was 27% and 31% in *35S*::*DCA1* plants and CK plants, respectively (Fig 7B). In contrast, there was no significant difference in stomatal density and guard cell size between *35S*::*DCA1* and CK plants (Fig 7C and S7B and S7C Fig). Following dehydration stress, *35S*::*DCA1* plants lost more water than CK plants (Fig 7D). We next investigated stomatal aperture following drought stress, and statistical analysis revealed that 14% of stomata were completely closed in *35S*::*DCA1* plants vs. 28% in CK plants, while 37% of stomata were completely open in *35S*::*DCA1* plants vs. only 27% in CK plants (S7F Fig). The percentage of partially open stomata was 49% and 45% in *35S*::*DCA1* plants and CK plants, respectively (S7F Fig). The ratio of completely closed stomata was 11% vs. 6% in *dca1* mutant and Ni (wild-type) plants, and 60% of stomata were completely open in the *dca1* mutant compared with 67% in wild-type plants (Fig 7E). The percentage of partially open stomata was similar in *dca1* mutant and wild-type plants (Fig 7E). At the same time, low magnification Cryo SEM images showed that no significant changes occurred in stomatal density in *dca1* plants (Fig 7F), and there were also no significant changes in guard cell size (S7D and S7E Fig). Meanwhile, the rate of water loss of detached leaves was lower in the *dca1* mutant plants than in wild-type plants (Fig 7G). When under drought stress, statistical analysis revealed that 17% of stomata were completely closed in Ni plants vs. 25% in *dca1* plants, while 38% of stomata were completely open in Ni plants vs. only 27% in *dca1* plants (S7G Fig). The percentage of partially open stomata was 44% and 48% in Ni plants and *dca1* plants, respectively (S7G Fig). Together these results suggest that the enhanced drought tolerance observed in the *dca1* mutant is mainly due to increased stomatal closure, which minimizes water loss.

### DCA1 Influenced H_2_O_2_ Content in Stomata via Prx24

**Fig 8:**
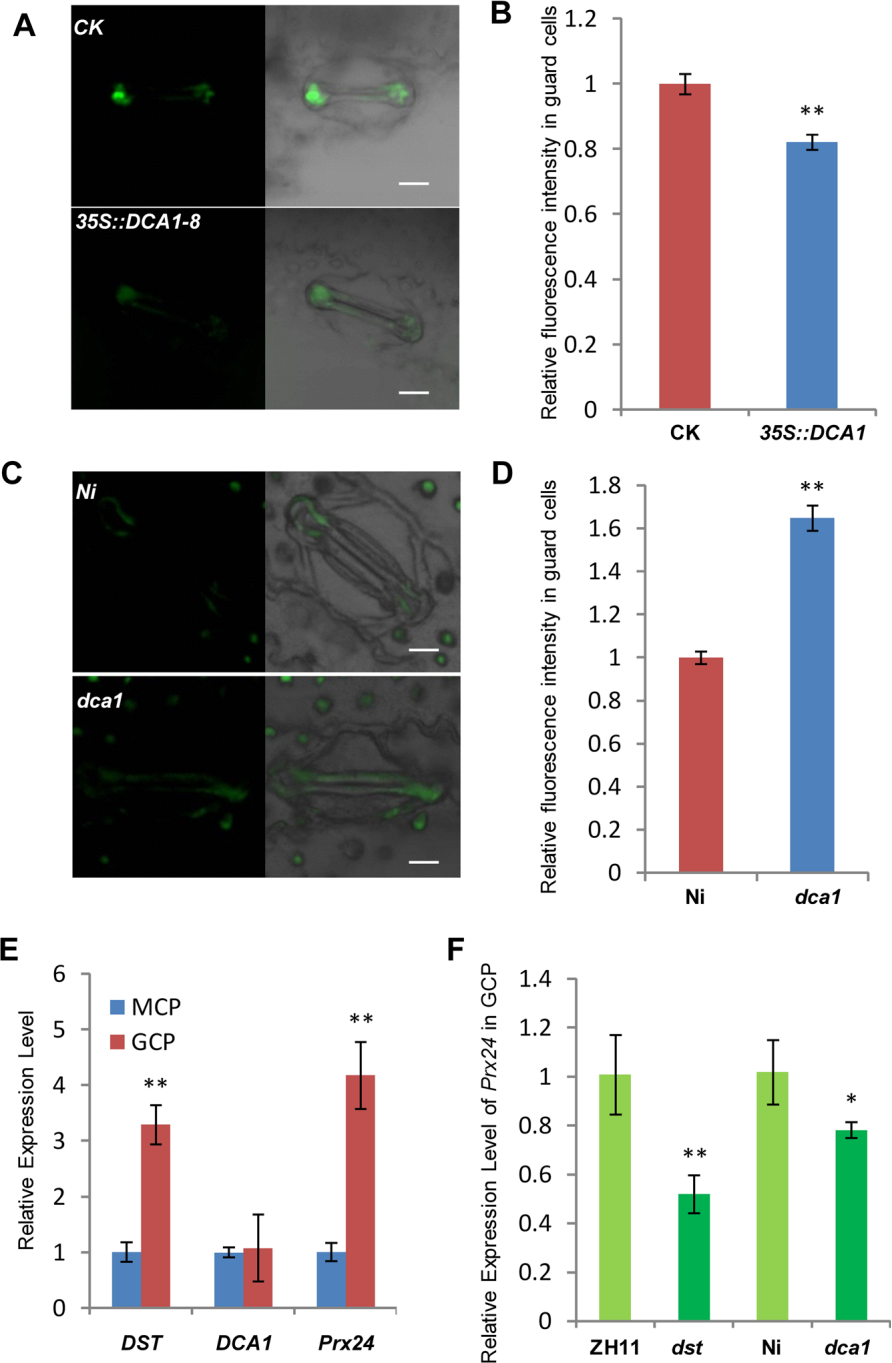
DCA1 influences H_2_O_2_ content in stomata. (A) H_2_O_2_ levels in guard cells of CK and *35S*::*DCA1-8* plants labeled with H_2_DCFDA. (B) Quantitative analysis of H_2_O_2_ production in the guard cells of CK and *35S*::*DCA1-8* plants (n = 8 leaves, ~10 stomata per leaf; 86 stomata for CK; 79 stomata for *35S*::*DCA1-8*). Data represent means ± s.e.m. (n = 8). (C) H_2_O_2_ production in guard cells of Ni and *dca1* plants labeled with H_2_DCFDA. (D) Quantitative analysis of H_2_O_2_ production in the guard cells of Ni and *dca1* plants (n = 8 leaves, ~10 stomata per leaf; 77 stomata for Ni; 92 stomata for *dca1*). Data represent means ± sem (n = 8). (E) Expression levels of *DST*, *DCA1* and *Prx24* in guard cell protoplasts (GCPs) and mesophyll cell protoplasts (MCPs) of ZH11 normalized against actin. Data represent means ± sd (n = 3). (F) Expression levels of *Prx24* in GCPs from ZH11, *dst*, Ni and *dca1*. Data represent means ± sd (n = 3). Significant differences were determined using the Student’s t-test (**P <0.01).

The phytohormone ABA is known to induce stomatal closure [23], and measurement of the endogenous ABA content revealed no significant difference between *dst* mutant and wild-type plants [21]. H_2_O_2_ can also induce leaf stomatal closure [16, 24, 25], and Huang *et al*. observed increased stomatal closure in the *dst* mutant as a result of H_2_O_2_ accumulation [21]. We therefore examined the H_2_O_2_ content in the stomata of *DCA1*-overexpressing and mutant plant leaves using the fluorescent dye 2’, 7’-dichlorodihydrofluorescein diacetate (H_2_DCFDA). We found that overexpression plants contained ~20% less H_2_O_2_ in their guard cells than did CK plants (Fig 8A and 8B). However, mutant plants accumulated more H_2_O_2_ in their guard cells than did Ni plants (Fig 8C and 8D). To explore the molecular mechanism behind this phenomenon, we isolated guard cell protoplasts (GCP) and mesophyll cell protoplasts (MCP) from ZH11, *dst*, Ni and *dca1* plants to examine the expression levels of *DCA1*, *DST* and *Prx24*. The results revealed higher levels of *Prx24* and *DST* expression in GCPs than MCPs of ZH11, but *DCA1* expression was not elevated (Fig 8E). *Prx24* was expressed at relatively lower levels in *dca1* mutant and *dst* mutant GCPs than in CK plants (Fig 8F), and since Prx24 functions as a ROS scavenger, these results indicated that the H_2_O_2_ content in stomata may be affected by *DCA1-DST*.

### DST Physically Interacts with Itself and Its Expression Is Affected by Abiotic Stress

**Fig 9:**
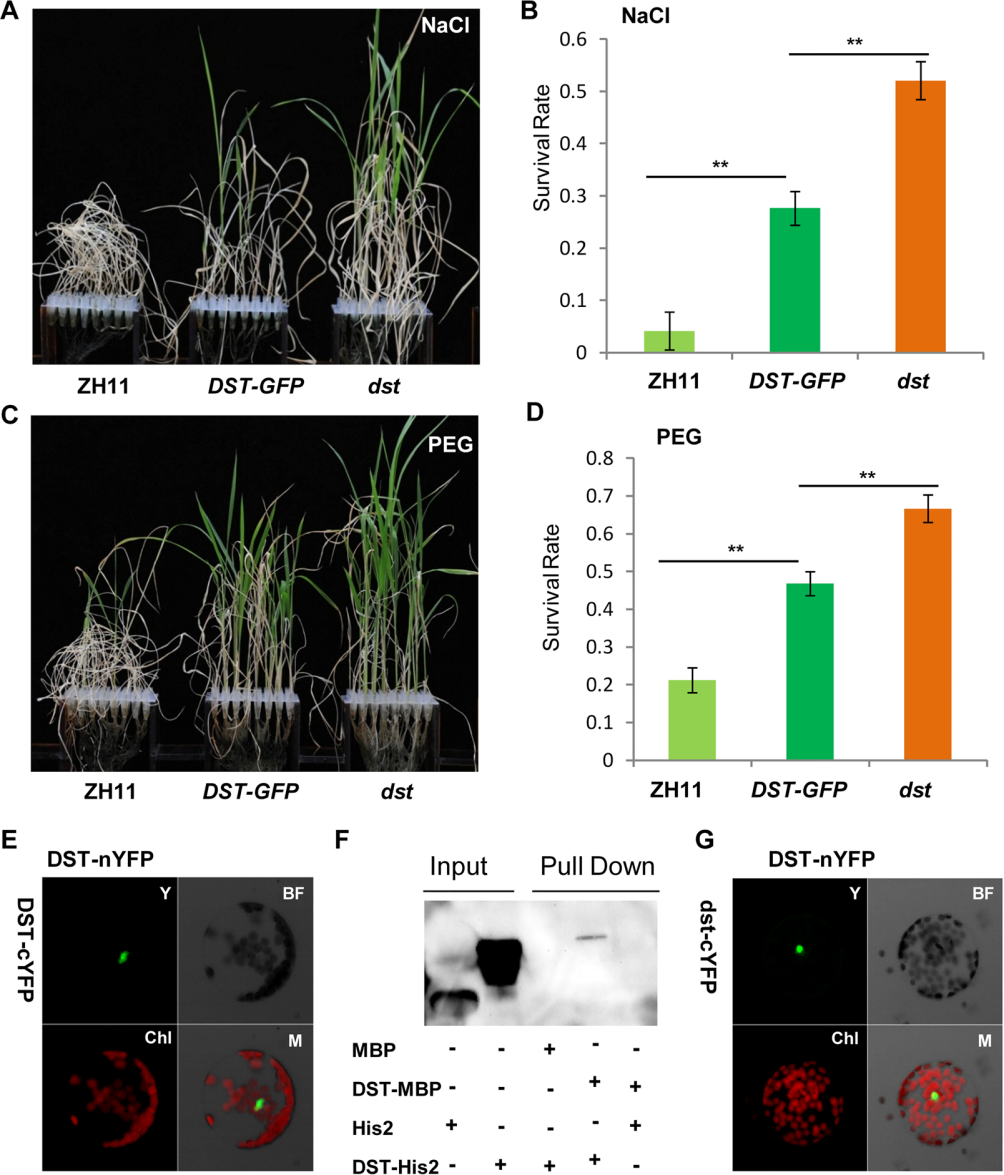
DST functions as a dimer. (A) 12-day-old ZH11, *DST-GFP* (*DST*:*pro*::*DST-GFP* in *dst* background) and *dst* seedlings treated with 100 mM NaCl for 10 days and recovered for 7 days. (B) Survival percentage after NaCl treatment (n = 3 biological replicates, 24 plants of each replicate). Data represent means ± sd (n = 3). (C) 12-day-old ZH11, *DST-GFP* and *dst* seedlings treated with 18% PEG for 12 days and recovered for 7 days. (D) Survival percentage after PEG treatment (n = 3 biological replicates, 24 plants of each replicate). Data represent means ± sd (n = 3). (E) BiFC assay in Arabidopsis protoplasts revealing DST dimerization. Y, YFP; BF, Bright Field; Chl, Chlorophyll; M, Merge. (F) *In vitro* pull-down of His2-tagged DST protein through MBP-tagged DST protein detected by immunoblotting with anti-His antibody. (G) Mutant isoform of DST do not affect dimerization, consistent with the BiFC assay results. Y, YFP; BF, Bright Field; Chl, Chlorophyll; M, Merge. Significant differences were determined using the Student’s t-test (**P <0.01).

While carefully observing the phenotypes of *DST*-complemented (*DSTpro*::*DST-GFP* in *dst*) plants under normal and stress conditions, we found that the *dst* mutant was not fully complemented. Specifically, the phenotype of *DSTpro*::*DST-GFP* under stress conditions was intermediate between those of wild-type and the *dst* mutant plants (Fig 9A–9D). We hypothesized that perhaps the mutated form of DST exhibiting reduced transcriptional activity could still function in the transcriptional complex. A BiFC assay in Arabidopsis protoplasts revealed that DST interacts with itself *in vivo* (Fig 9E), and an *in vitro* pull-down assay showed that MBP-DST was able to pull down the His2-DST protein (Fig 9F and S8A Fig). Meanwhile, the mutant site of DST did not influence its dimerization (Fig 9G). These results confirm that our hypothesis was correct, and indicate that DST functions as a dimer. In addition, the complex containing the mutant isoform could not fully execute its transactivation function, which may explain why the *dst* mutant was not fully complemented. We also examined whether DCA1 functions in the same manner with DST, and pull-down assays revealed that it does not (S8B Fig). Further analysis revealed that the DST-DST interaction was negatively influenced by stress in Arabidopsis protoplast cells (S9A, S9B and S9E Fig). This may be due to decreased levels of DST monomer or decreased DST dimerization. To investigate further, *DST* self-promoter promoted DST-nYFP and DST-cYFP were cotransformed into Arabidopsis protoplasts and cultured for 9 h under normal conditions. Half of the cells were maintained in normal conditions, while the other half were treated with NaCl and cycloheximide (CHX), an inhibitor of protein biosynthesis. After 3 h, the relative fluorescence ratios of the cells were similar (S9C, S9D and S9F Fig). These results demonstrate that DST was inhibited by stress, but not degraded or dissociated into monomeric form, and this was verified by qRT-PCR experiments (S2B and S2C Fig). These results, when combined with those from the EMSA experiments (Fig 6E), indicate that DST and DCA1 may form a putative heterotetrameric complex that participates in gene regulation.

## Discussion

In this study, we identified the DST interacting protein DCA1. Overexpression of *DCA1* increased the plant’s sensitivity to abiotic stress, and *35S*::*DST* plants were also highly sensitive to salt and drought stress. Knockdown of *DCA1* improved the tolerance to stress, as also observed in the *dst* mutant. These results indicate that DCA1 has a positive effect on the transcriptional activity of DST, which was confirmed by dual luciferase assays. The qRT-PCR results showed that *DST* was suppressed under stress conditions while *DCA1* was induced (Fig 2E and 2F and S2B and S2C Fig). Hsu, *et al*. (2014) identified *ZFP34*, a homolog of *DCA1*, and discovered that *ZFP34* participates in the heat stress response. Overexpression of this gene in rice and its homolog in Arabidopsis increased stomatal opening, and *ZFP34* mutants exhibited decreased stomatal opening in both rice and Arabidopsis [22]. Heat stress is usually accompanied by drought, since transpiration is induced when plants experience high temperatures. *DCA1* expression is therefore likely to be upregulated by drought, salt and heat stress. Water loss caused by increased transpiration may aggravate the impact of drought stress, and plants have evolved very sophisticated molecular mechanisms to overcome this paradoxical phenomenon. One possible molecular mechanism may be the *DCA1-DST* pathway, since *DCA1* may induce the opening of stomata to reduce the internal temperature during heat stress. At the same time downregulation of *DST* may cause closure of stomata to prevent excessive moisture loss. This seemingly contradictory phenomenon ensures that the plant can adapt to various environmental changes in different ways, which builds robustness into the plant responses to abiotic stress.

The DCA1 protein contains two zinc ion binding sites, a CHY zinc finger domain and a RING-H2 domain. The CHY domain is highly conserved among homologous proteins from different species, but the RING-H2 domain is not highly conserved. Therefore, the CHY domain may be the major functional part of this protein family, but the exact function was unknown until now. In the present study, we found that DCA1 functions as a transcriptional co-activator. However, the means by which DCA1 promotes the activity of DST is not yet elucidated. DCA1 may alter the conformation of DST through the protein-protein interaction, or may help DST to recruit other transcription initiation factors such as RNA polymerase, or may even function as a regulator of chromatin structure. Many RING finger domain proteins function as ubiquitin ligases, although many are also involved in other roles such as Breast Cancer 1 (BRCA1) which participates in transcriptional regulation, DNA damage repair and chromatin remodeling [26, 27]. Mex-3 homolog D (MEX3D) is an RNA binding protein in *C*. *elegans* that has a negative regulatory action on *Bcl-2* expression at the posttranscriptional level [28], and peroxisome biogenesis factor 10 (PEX10) is involved in import of peroxisomal matrix proteins [29]. Thus, DCA1-like proteins may also perform a range of different functions.

**Fig 10:**
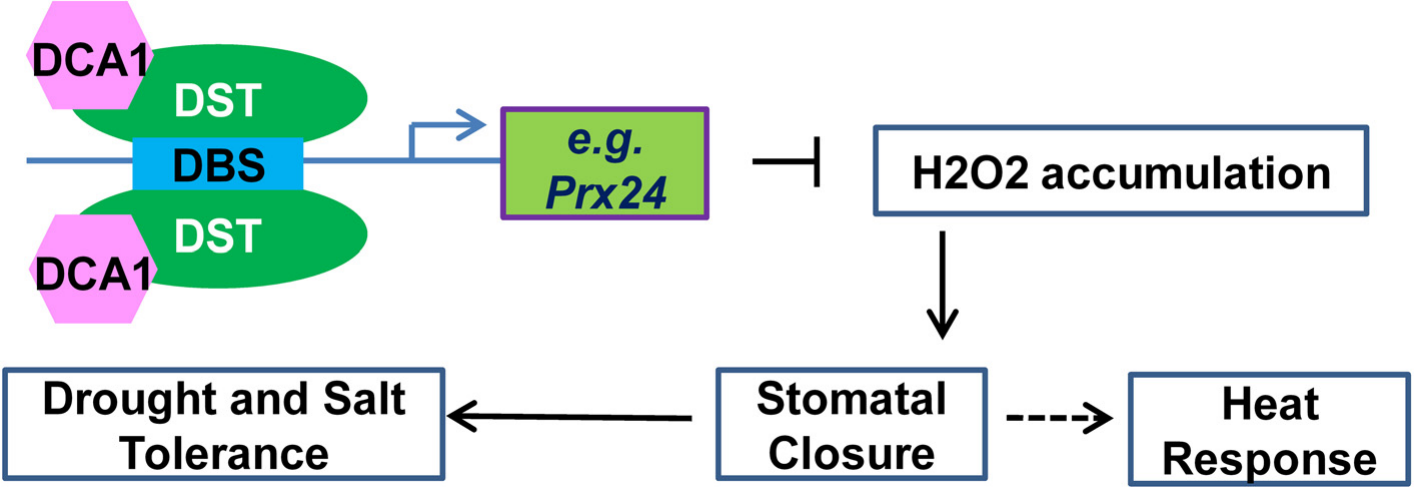
Working model for the role of the DCA1-DST transcriptional regulation complex in plants. The DCA1 and DST heterotetrameric complex binds to the DBS element in the promoters of genes associated with H_2_O_2_ homeostasis in stomata such as *Prx24*, thereby influencing stomatal closure and ultimately regulating abiotic stress response.

Previous studies focusing on H_2_O_2_ signalling in guard cells during stress conditions have indicated that NADPH oxidases such as *Atrboh* D and *Atrboh* H may play major roles in this process [10]. Indeed, the *atrbohD/F* double mutant exhibited reduced stomatal closure and ROS production compared with wild-type plants. We previously identified the transcription factor DST that binds to the DBS element to regulate the expression of H_2_O_2_ metabolism-associated genes in stomata, and extended this work by identifying the DST interacting CHY zinc finger protein DCA1 in the present study. This transcriptional complex appears to regulate the expression of *Prx 24*, an H_2_O_2_ scavenger that we found to be more highly expressed in guard cells than in mesophyll cells. Our results reveal how plants prolong H_2_O_2_ signalling by reducing the catabolism of H_2_O_2_ through the DCA1-DST-Prx24 pathway. Based on these results, we propose a simple model to explain the role of the DCA1-DST-Prx24 pathway in regulating the status of guard cells and stress tolerance. In this model, DCA1 functions as an interacting co-activator of DST to form a heterotetrameric complex that regulates H_2_O_2_ homeostasis and stomatal aperture, ultimately affecting stress tolerance (Fig 10).

Drought and salinity have had devastating effects on food production throughout human history, and the problems are increasing with the growing population. During 2010–2012, drought occurred on most continents, and millions of hectares of crops were destroyed, from wheat in Europe to corn in the US to rice in China [30, 31]. In 2014, the state of California in the US suffered its worst drought on record [32]. Engineering the stomatal response to improve water use efficiency represents a powerful tool for enhancing drought tolerance in plants. Indeed, the constitutive reduction in stomatal opening in the rice *dca1* and *dst* mutant enhances drought tolerance. Given the high conservation of DCA1 among plants, our research suggests that inhibiting the functional association of DCA1-DST may provide one route to unlocking improved drought and salt tolerance in a variety of crops.

## Materials and Methods

### Plant Materials and Growth Conditions

All rice genetic stocks used in this study were in the Zhonghua 11 (ZH11) *japonica* variety background except for *dca1*, which is in the Nipponbare (*japonica* variety) background. *35S*::*DCA1* plants were generated via transformation with a construct produced by inserting an amplified ORF fragment containing *DCA1* from ZH11 cDNA between the CaMV35S promoter and the 3’OCS terminator. *35S*::*DST* plants were generated using a similar method. The *dca1* mutant (NF7038) was obtained from the Rice Genome Resource Center.

We generated knockdown transgenic plants using an artificial microRNA (designed by http://wmd3.weigelworld.org/cgi-bin/webapp.cgi). Target site on *DCA1* was 5’- acgtgatgagagcgcatcatc-3’. The construction method was modified from Norman Warthmann *et al*. [33]. For the miRNA construct, three modification PCRs were performed with primers G-11491 + DmiR II, DmiR I + DmiR IV and DmiR III + G-11494 on ZH11 cDNA as template, yielding three fragments, respectively (S1 Table). The three resulting fragments were gel purified (Qiagen) and then fused by one PCR with the two flanking primers G-11491 + G-11494 on a mixture of 1 ul from each of the previous three PCRs as template. The fusion product was again gel purified (Qiagen), cloned into the overexpression vector to generate *DCA1* knockdown transgenic plants.

Seeds were submerged in water at room temperature for 48 h, followed by germination for 18 h at 37°C. Seeds were then sown in a bottomless 96-well plate that was placed in a container of Yoshida’s culture solution and incubated in a growth chamber under a 13 h light (25°C)/11 h dark (23°C) photoperiod. For salt treatment, 20-day-old seedlings were transferred to a culture solution containing 100 mM NaCl. For PEG treatment, 20-day-old seedlings were transferred to a culture solution containing 18% (w/v) PEG4000. For soil drought experiments, 2-week-old seedlings that were cultured in the growth chamber were transplanted to containers with soil and grown in a greenhouse at 24–30°C and 50–60% relative humidity. About 50 days later, water was removed from the containers for the dehydration treatment.

### Yeast Two-Hybrid Assays

Screening for DST interacting proteins was performed according to the manufacturer’s instructions (Clontech). Briefly, libraries were prepared using total RNA extracted from ZH11 leaves that were approximately 20 days old. The DST fragment lacking the self-activation domain (amino acids 1–72) was cloned into the pGBKT7 BD vector to generate pGBKT7/△72 (S1 Table). Screening was performed on SD/–Leu/–Trp medium containing 2.5 mM 3-aminotriazole (3-AT). To confirm positive interactions, full-length ORFs of each target gene were cloned into the pGADT7 AD vector and cotransformed with pGBKT7/△72 into Y2HGold Competent Cells. To verify positive interactions, the full-length *DST* ORF was cloned into the pGADT7 AD vector, and target genes were cloned into the pGBKT7 BD vector, and Y2HGold Competent Cells were cotransformed with 100 ng of each vector pair. Cells were grown at 30°C for 3 days on SD/–Ade/–His/–Leu/–Trp medium containing 1–10 mM 3-AT (depending on the bait—prey combination).

### Transient Expression

DST (GQ178286) and DCA1 (Os10g0456800) coding regions were cloned into YFP expression vectors, BiFC vectors or dual luciferase system vectors. For subcellular localization experiments, 10 μg of YFP-DST, YFP-DCA1 or YFP expression plasmid was transformed into Arabidopsis protoplasts and observed under a confocal microscope (Carl Zeiss). For BiFC experiments, 5 μg each of YFP-N- and YFP-C-tagged protein expression vector was cotransformed. For the dual luciferase transient transcription activity assay, a 7× GAL4 binding sequence was inserted into the pGreenII 0800-LUC vector to generate the LUC reporter construct. The coding sequence of DST was inserted into pGreen GAL4BD to produce the effector construct, and DCA1 was inserted into pGreenII 62-SK (S1 Table). Firefly LUC and Renilla luciferase (REN) activities were measured using the dual luciferase reporter assay system (Promega). The LUC:REN ratio represents transcriptional activity.

### Protein Expression

The DST ORF was inserted into pET32a, pCold TF and pMAL-c5x vectors. The DCA1 coding sequence was inserted into pCold TF and pMAL-c5x (S1 Table). The tag in pET32a was designated His1 and that in pCold TF was designated His2. The pMAL-c5x constructs were transformed into TB1 competent cells, and pET32a and pCold TF vectors were transformed into DE3 competent cell.

### Pull-Down Assay

The pull-down assay was performed according to the instructions for the MagneGST Pull-Down System (Promega) with some modifications. Briefly, *E*. *coli* cells expressing DST-MBP and MBP were lysed with BugBuster Protein Extraction Reagents (Novagen) and centrifuged. The supernatant was incubated with Anti-MBP Magnetic Beads (NEB) for 30 min at 4°C and washed five times with MBP column buffer. Then, supernatants containing DCA1-His2 and His2 proteins were incubated with approximately the same amount of MBP and DST-MBP binding beads for 2 h at 4°C. The magnetic beads were then washed five times with MBP column buffer. The mixture was resuspended in SDS loading buffer, boiled for 3 min, separated by 10% SDS-PAGE and immunoblotted with anti-MBP antibody for target proteins and anti-His antibody for pull-down proteins (CWbiotech).

### RNA Extraction and Real-Time PCR Analysis

Leaves from 2-week-old seedlings were harvested and total RNA was extracted using TRIzol reagent (Invitrogen) according to the manufacturer’s instructions. 1–2 μg of total RNA was used for cDNA synthesis with oligo (dT) primer and Superscript Reverse Transcriptase (Invitrogen). Quantitative RT-PCR was performed using FastStart Universal SYBR Green Master (Roche) and an ABI 7300 Real-time PCR System (Applied Biosystems). The expression of *DST*, *DCA1* and *Prx*24 was normalized against *actin*. Oligonucleotide primers are listed in S1 Table.

### Cryo Scanning Electron Microscopy (Cryo SEM)

Leaves from 20-day-old seedlings grown under normal conditions or treated with 12% PEG for 6 hours were detached and frozen in liquid nitrogen. Images of stomata were obtained using a Cryo (QUORUM) scanning electron microscopy (JEOL) system (Cryo SEM).

### Measurement of H_2_O_2_ in Guard Cells

H_2_O_2_ production in guard cells was detected using H_2_DCFDA (Molecular Probes) as described by Huang [21]. Epidermal strips were peeled from leaves of 20-day-old rice seedlings using tissue forceps, washed with loading buffer (10 mM Tris-HCl, 50 mM KCl, pH 7.2) and incubated in staining buffer (loading buffer containing 50 mM H2DCFDA) for 10 min at 25°C in the dark. Strips were washed twice with distilled water to remove excess staining buffer, and fluorescence was measured using a confocal laser-scanning microscope (Olympus) with excitation at 488 nm and emission at 525 nm. All images were taken under identical conditions. To quantify fluorescence, the guard cell region was selected and the mean fluorescence value was calculated using the microscope’s software. Each sample contained at least eight independent leaves, and approximately 10 randomly selected stomata were analyzed per leaf.

### Isolation of Mesophyll Cell Protoplasts (MCP) and Guard Cell Protoplasts (GCP) from Rice Leaves

30 fully expanded one-month-old rice leaves were cut into ~0.3 cm sections and blended with 200 mL Lysis buffer (5 mM CaCl2, 0.5 mM ascorbic acid, 0.1% PVP-40, 10 mM MES, pH 6.0) for 90 s using a blender (Waring blender model LB20ES). The blended material was filtered through a 200 μm nylon mesh, washed once with 200 ml ddH2O, suspended in 100 ml suspension buffer (0.25 M mannitol, 1mM CaCl2, 0.5mM ascorbic acid), filtered through a 30 μm nylon mesh and transferred into a flask with 25 mL of an enzyme mixture containing 0.7% Cellulysin cellulase, *Trichoderma viride* (Sigma), 0.1% (w/v) PVP-40, 0.25% BSA in 55% (v/v) basic medium (5 mM MES, 0.5 mM CaCl2, 0.5 mM MgCl2, 10 μM KH2PO4, 0.5 mM ascorbic acid (Sigma), 0.55 M sorbitol, pH 5.5), and 45% (v/v) deionized water. The flask was placed in a shaking water bath at 24°C with the shaking speed set to 120 rpm. After 3 h of digestion, 75 mL basic medium was added, and the mixture was shaken for an additional 5 min. The mixture was filtered through a 70 μm nylon mesh and washed twice with 50 mL basic medium. MCPs were collected by centrifuging the filtrate at 200 g for 5 min. Epidermal fragments were transferred to another flask with 50 mL of basic medium (1.5% Cellulase RS (Yakult, Japan), 0.02% Pectolyase Y23 (Yakult, Japan), 0.25% BSA, pH 5.5) and incubated at 24°C in a shaking water bath (100 rpm). After incubation for 3 h, the mixture was filtered through a 30 μm nylon mesh, washed twice with 50 mL basic medium, and GCPs were collected at 200 g for 5 min. Two different transcription inhibitors, actinomycin D and cordycepin, were used to prevent changes in gene expression during protoplast isolation.

### EMSA

EMSA was performed as described by Huang [21] using His1 tag, DST-His1, His2 tag and DCA1-His2 recombinant fusion proteins. Biotin-labeled oligonucleotides were obtained by PCR amplification using biotin-labeled primers (Invitrogen). The binding reactions (containing biotin-labeled DNA and different combinations of proteins) were performed with a Light Shift Chemiluminescent EMSA kit (Pierce) according to the manufacturer’s instructions. Gel electrophoresis was performed on a 10% native polyacrylamide gel (79:1 acryl/bis). After blotting onto a nylon membrane, DNA was attached to the membrane using a UV light cross-linker instrument. Blots were imaged using a Tanon 5200 imaging system (bioTanon).

### Sequence Alignment and Phylogenic Analysis

Peptide sequences were obtained from Phytozome (Phytozome v9.1, http://www.phytozome.net/) and sequence alignment was performed using the Clustal Omega program (http://www.ebi.ac.uk/Tools/msa/clustalo/).

## Supporting Information

S1 Fig DCA1 and homologous proteins from different species. (A) Domain structure of DCA1. (B) Conservation Analysis of DCA1 homologs from Different Species. Zma, *Z*. *mays*; Ath, *Arabidopsis thaliana*; Sbi, *Sorghum bicolor*; Osa, *Oryza sativa*. Red triangles indicate the CHY zinc finger; black triangles indicate the C_3_H_2_C_3_ domain.
(PDF)

S2 Fig Expression of *DST*. (A) Expression of *DST* in different tissues of ZH11. (B, C) Relative expression levels of *DST* in the leaves of plants treated with NaCl and PEG. Data represent means ± sd (n = 3).
(PDF)

S3 Fig Relative chlorophyll content and fresh weight following drought stress treatment. (A) Relative chlorophyll content of CK, *35S*::*DCA1-8* and *35S*::*DST-1* after stress treatment. (B) Relative chlorophyll content of Ni and *dca1* after stress treatment (n = 3 groups, each containing 3 leaves). Data represent means ± sd. (C) Relative fresh weight of CK, *35S*::*DCA1-8* and *35S*::*DST-1* after stress treatment. (D) Relative fresh weight of Ni and *dca1* after stress treatment (n = 3 groups, each containing 3 plants). Data represent means ± sd. Significant differences were determined using the Student’s t-test (**P <0.01).
(PDF)

S4 Fig Artificial microRNA knockdown of *DCA1* increases stress tolerance in rice. (A) Southern blot of Ni and *dca1*. (B) CK (vector in ZH11), *DmiR-1* and *DmiR-2* grown under normal conditions for 12 days (left), 12-day-old seedlings transferred to 100 mM NaCl for 11 days and recovered for 7 days (middle), or transferred to 18% PEG for 12 days and recovered for 7 days (right). (C) Expression levels of *DCA1* normalized against actin. Data represent means ± sd (n = 3). (D) Expression levels of *Prx24*. Data represent means ± sd (n = 3). (E) Relative survival rates following NaCl treatment (n = 3 biological replicates, 24 plants of each replicate). Data represent means ± sd. (F) Relative survival rates following PEG treatment (n = 3 biological replicates, 24 plants of each replicate). Data represent means ± sd. Significant differences were determined using the Student’s t-test (**P <0.01).
(PDF)

S5 Fig Relative expression levels of *DST* in *35S*::*DCA1* and *35S*::*DST* plants. (A, B) Relative expression level of *DST* in *35S*::*DST* and *35S*::*DCA1* plants grown under normal conditions. Samples were collected from 18-day-old seedlings. Data represent means ± sd (n = 3). (C, D) Seed setting on the main panicle of Ni and *dca1* after soil dehydration treatment. Data represent means ± sd (n = 8). Significant differences were determined using the Student’s t-test (**P <0.01).
(PDF)

S6 Fig Expression and phenotype of *Prx24*. (A, B) Real-time PCR quantification of the DST downstream target gene *Prx24* in CK, *35S*::*DST-1* and *35S*::*DST-2* (A) and CK, *35S*::*DCA1-7* and *35S*::*DCA1-8* (B). Data represent means ± sd (n = 3). (C) CK (vector in ZH11), *35S*::*Prx24-2* and *35S*::*Prx24-4* seedlings grown under normal conditions for 12 days (left), transferred to 100 mM NaCl for 9 days and recovered for 7 days (middle), or transferred to 18% PEG for 11 days and recovered for 7 days (right). (D) EMSA of DST and DCA1 proteins with biotin-tagged Pro24 (Bio-Pro24), a DST binding site-containing *Prx24*-promoter DNA sequence. The triangle indicates the shifted band. FP, free probe. Significant differences were determined using the Student’s t-test (**P <0.01).
(PDF)

S7 Fig *DCA1* affects stomatal aperture but not stomatal development. (A) Cryo scanning electron microscopy (Cryo SEM) images of three levels of stomatal opening of Ni. (B, C, D, E) Overview of stomatal status of CK (B), *35S*::*DCA1-8* (C), Ni (D) and *dca1* (E) plants under low magnification. (F, G) Percentage of the three levels of stomatal opening in CK and *35S*::*DCA1-8* (F) and Ni and *dca1* (G) under drought conditions (12% PEG for 6 h, n = 8 samples, 10 stomata per sample). Data represent means ± sd. Significant differences were determined using the Student’s t-test (*P<0.05, **P <0.01).
(PDF)

S8 Fig *In vitro* dimerization of DST and DCA1. (A) *In vitro* pull-down of His2-tagged DST through MBP-tagged DST detected by immunoblotting with anti-MBP antibody. (B) *In vitro* pull-down of His2-tagged DCA1 through MBP-tagged DCA1 detected by immunoblotting with anti-His antibody.
(PDF)

S9 Fig Response of DST to stress signals. YFP fluorescence in the BiFC assay indicates dimerization of DST. (A, B) NaCl treatment reduces the dimeric form of DST (B) compared with normal conditions (A). (C, D) The dimeric form of DST is not influenced by NaCl treatment with cycloheximide (CHX) (D) compared with normal conditions (C). (E) Quantitative statistical analysis of (A) and (B) (n = 3 biological replicates, each containing 50 cells). Data represent means ± sd (n = 3). (F) Quantitative statistical analysis of (C) and (D) (n = 3 biological replicates, each containing 50 cells). Data represent means ± sd (n = 3). Significant differences were determined using the Student’s t-test (**P <0.01).
(PDF)

S1 Table Oligonucleotides used in this study. (PDF)
