# Supplementary material for: DCA1 Acts as a Transcriptional Co-activator of DST and Contributes to Drought and Salt Tolerance in Rice
Source: PLoS Genet. 2015 Oct 23;11(10):e1005617. doi: 10.1371/journal.pgen.1005617 (PMC4619773; doi:10.1371/journal.pgen.1005617)
Supplement: S1 Fig — (A) Domain structure of DCA1. (B) Conservation Analysis of DCA1 homologs from Different Species. Zma, Z. mays; Ath, Arabidopsis thaliana; Sbi, Sorghum bicolor; Osa, Oryza sativa. Red triangles indicate the CHY zinc finger; black triangles indicate the C3H2C3 domain. (PDF) [file pgen.1005617.s001.pdf]

A

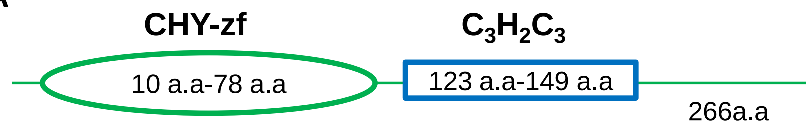

B

```
Zma:GRMZM2G052034_T02     FGCKHYRRRCRIRAPCCGDVFHCRHCHNESTK-------DGHELDRHDVQSVICLVCDTE
Ath:AT3G62970             FGCEHYKRRCKIRAPCCNLIFSCRHCHNDSANSLP-DPKERHDLVRQNVKQVVCSICQTE
Ath:AT5G18650             FGCKHYKRRCQIRAPCCNEVFDCRHCHNESTSTLR-NIYDRHDLVRQDVKQVICSVCDTE
Sbi:Sb01g035440           FGCKHYRRRCRIRAPCCGDVFHCRHCHNESTK-------DGHELDRHAVQSVICLVCDTE
Osa:LOC_Os03g22680.1      FGCKHYRRRCRIRAPCCNDVFHCRHCHNESTK-------DGHELDRHAVESVICLVCDTE
Ath:AT5G25560             YGCPHYRRRCCIRAPCCNEIFGCHHCHYEAKNNINVDQKQRHDIPRHQVEQVICLLCGTE
Ath:AT5G22920             YGCSHYRRRCKIRAPCCDEIFDCRHCHNEAKDSLHIEQHHRHELPRHEVSKVICSLCETE
Osa:DCA1                  HGCEHYTRGCRIRAPCCGEVFGCRHCHNEAKNSLEIHLNDRHEIPRHEIKKVICSLCDKE
Sbi:Sb01g047160           YGCVHYRRKCKIRAPCCGEVFDCRHCHNEAKDSLEVSIQDRHVVPRHDIKLVICSLCNKE
Osa:LOC_Os03g05270        YGCVHYRRKCKIRAPCCGEIFDCRHCHNEAKDSLEVSISDRHEIPRHEIKLVICSLCNKE
                          .** ** * * ******  :* *:*** :: .       . * : *: :. *:* :* .*

Zma:GRMZM2G052034_T02     QPIAQVCCNCGVCMGEYFCAACNFLDDDVDKEQFHCDDCGICSRRKGELLPL--------
Ath:AT3G62970             QEVAKVCSNCGVNMGEYFCDICKFFDDDISKEQFHCDDCGICRVGGRDKFFHCQNCGACY
Ath:AT5G18650             QPAAQVCSNCGVNMGEYFCSICIFYDDDTEKQQFHCDDCGICRVGGRENFFHCKKCGSCY
Sbi:Sb01g035440           QPIAQVCCNCGVCMGEYFCRKCNFLDDDVDKEQFHCDDCGICRVGGKDNFFHCQKCGSCY
Osa:LOC_Os03g22680.1      QPVAQVCYNCGVCMGEYFCSACKFFDDDVDREHFHCQDCGICRVGGKDNFFHCEKCGSCY
Ath:AT5G25560             QEVGQICIHCGVCMGKYFCKVCKLYDDDTSKKQYHCDGCGICRIGGRENFFHCYKCGCCY
Ath:AT5G22920             QDVQQNCSNCGVCMGKYFCSKCKFFDDDLSKKQYHCDECGICRTGGEENFFHCKRCRCCY
Osa:DCA1                  QDVQQYCSGCGACMGKYFCEKCNFFDDDVSKNQYHCDGCGICRTGGVDKFFHCDKCGCCY
Sbi:Sb01g047160           QDVQQDCSNCGACLGKYFCAKCNFFDDDVSKNQFHCDGCGICRTGGAENFYHCDKCGCCY
Osa:LOC_Os03g05270        QDVQQDCSNCGACLGKYFCAKCNFYDDDVSKNQFHCDGCGICRTGGAENFFHCDKCGCCY
                          *   : *  **. :*:***  * : *** .::::**: ****     : :

Zma:GRMZM2G052034_T02     -------------------PKVWILLLDDPSRQALLHR------ELDEEQL-PHLLRVPV
Ath:AT3G62970             GMGLRDKHSCIENSTKNSCPVCYEYLFDSVKAAHVMKCGHTMHMDCFEQMINENQYRCPI
Ath:AT5G18650             AVGLRNNHRCVENSMRHHCPICYEYLFDSLKDTNVMKCGHTMHVECYNEMIKRDKFCCPI
Sbi:Sb01g035440           STTLRDKHCCIENSMKNNCPICYEYMFDSLRETSVLRCGHTMHLHCFHEMLKHDKFSCPI
Osa:LOC_Os03g22680.1      SVSLRDKHCCIENSMKNNCPICYEYLFDSLRETSVLRCGHTMHLQCFHEMLKHDKFSCPI
Ath:AT5G25560             SILLKNGHPCVEGAMHHDCPICFEFLFESRNDVTVLPCGHTIHQKCLEEMRDHYQYACPL
Ath:AT5G22920             SKIMEDKHQCVEGAMHHNCPVCFEYLFDSTRDITVLRCGHTMHLECTKDMGLHNRYTCPV
Osa:DCA1                  SNVLRDSHHCVEGAMHHNCPVCFEYLFDSTKDISVLHCGHTIHLECLNVMRAHHHFACPV
Sbi:Sb01g047160           TSLLKDSHRCVDRAMHNNCPVCIEYLFDSTKAISVLHCGHTIHLECLYEMRAHQQFSCPV
Osa:LOC_Os03g05270        SYVLKDSHHCVERAMHHNCPVCFEYLFDSTKDISALHCGHTIHLECLYEMRSHQQFSCPV
                                             *     :::.      :        .             *:

Zma:GRMZM2G052034_T02     RF----AEGDVGAPLRPHHAPAVLPRDVEARQVLVPHMRHAHLRHGQVLQGPRRRVLIDA
Ath:AT3G62970             CAKSMVDMSPSWHLLDFEISATEMPVEYKF-EV-----------------------S---
Ath:AT5G18650             CSRSVIDMSKTWQRLDEEIEATAMPSDYRDKKV-----------------------W---
Sbi:Sb01g035440           CATSIFDMDKFFKALDAEMEASY---FYMG-KG-----------------------W---
Osa:LOC_Os03g22680.1      CSMPIFDMDKFLRALDAEIEANMLHIDYMG-KG-----------------------W---
Ath:AT5G25560             CSKSVCDMSKVWEKFDMEIAATPMPEPYQNRMV-----------------------Q---
Ath:AT5G22920             CSKSICDMSNLWKKLDEEVAAYPMPKMYENKMV-----------------------W---
Osa:DCA1                  CSRSACDMSDAWKKLDEEVAATPMPEFYQKKMI-----------------------W---
Sbi:Sb01g047160           CLRSACNMSDIWQKLDQEVAASPMPAIYQKKMI-----------------------W---
Osa:LOC_Os03g05270        CLRSACDMSHAWQKLDQEVAASPMPVIYQKKMI-----------------------W---
```
