# Supplementary figures and images for: DCA1 Acts as a Transcriptional Co-activator of DST and Contributes to Drought and Salt Tolerance in Rice
Source: PLoS Genet. 2015 Oct 23;11(10):e1005617. doi: 10.1371/journal.pgen.1005617 (PMC4619773; doi:10.1371/journal.pgen.1005617)

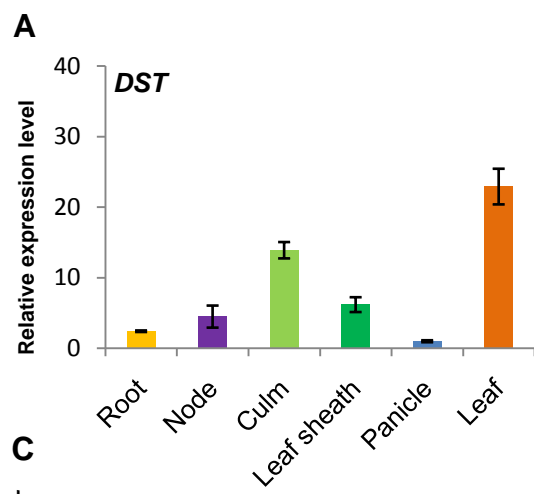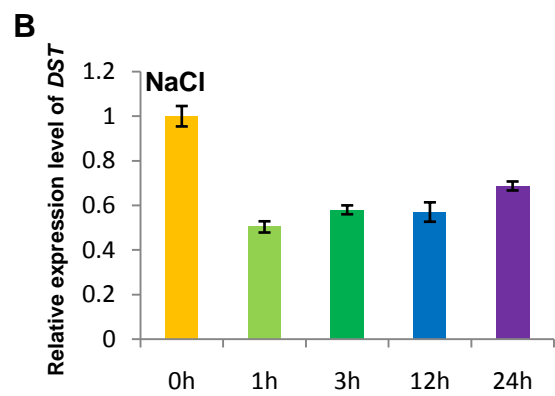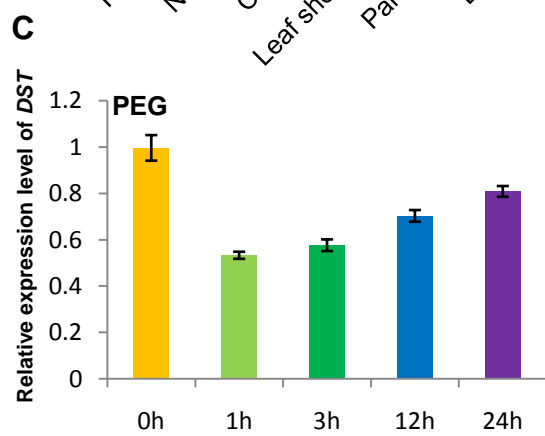

Supplement: S2 Fig — (A) Expression of DST in different tissues of ZH11. (B, C) Relative expression levels of DST in the leaves of plants treated with NaCl and PEG. Data represent means ± sd (n = 3). (PDF) [file pgen.1005617.s002.pdf]

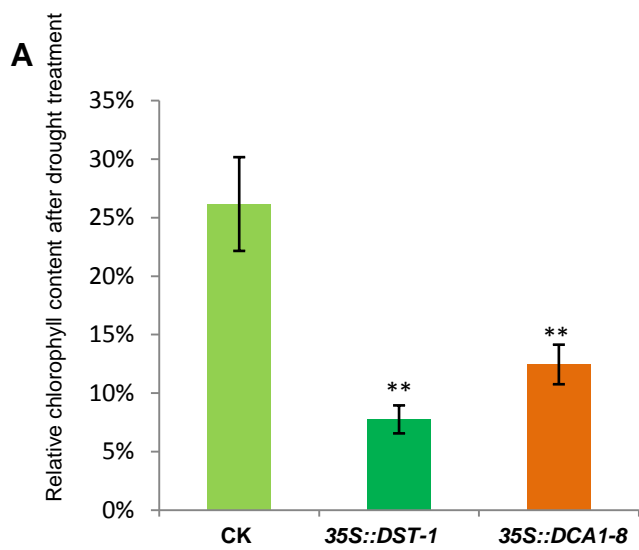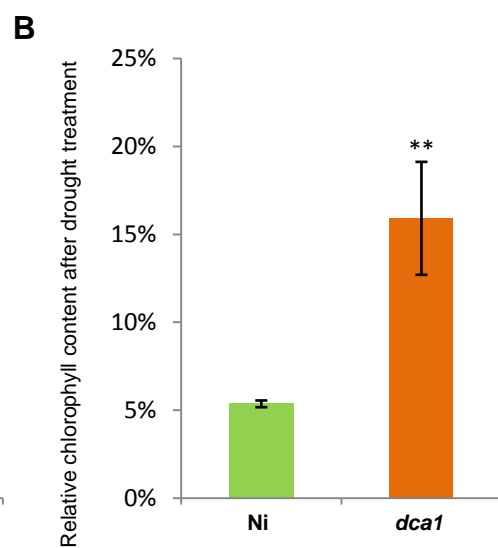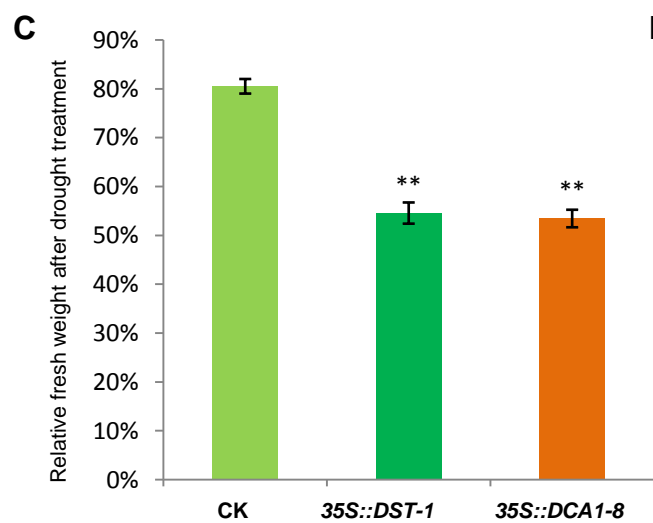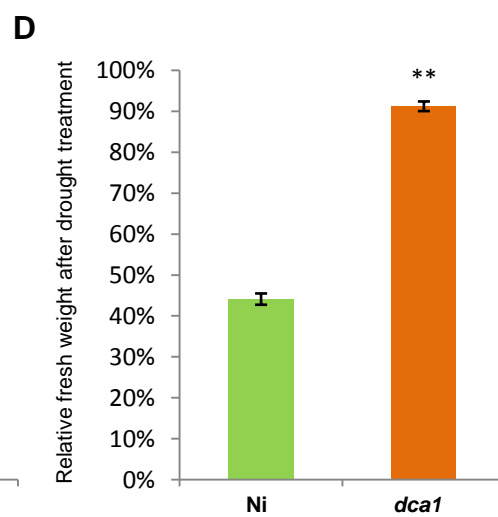

Supplement: S3 Fig — (A) Relative chlorophyll content of CK, 35S::DCA1-8 and 35S::DST-1 after stress treatment. (B) Relative chlorophyll content of Ni and dca1 after stress treatment (n = 3 groups, each containing 3 leaves). Data represent means ± sd. (C) Relative fresh weight of CK, 35S::DCA1-8 and 35S::DST-1 after stress treatment. (D) Relative fresh weight of Ni and dca1 after stress treatment (n = 3 groups, each containing 3 plants). Data represent means ± sd. Significant differences were determined using the Student’s t-test (**P <0.01). (PDF) [file pgen.1005617.s003.pdf]

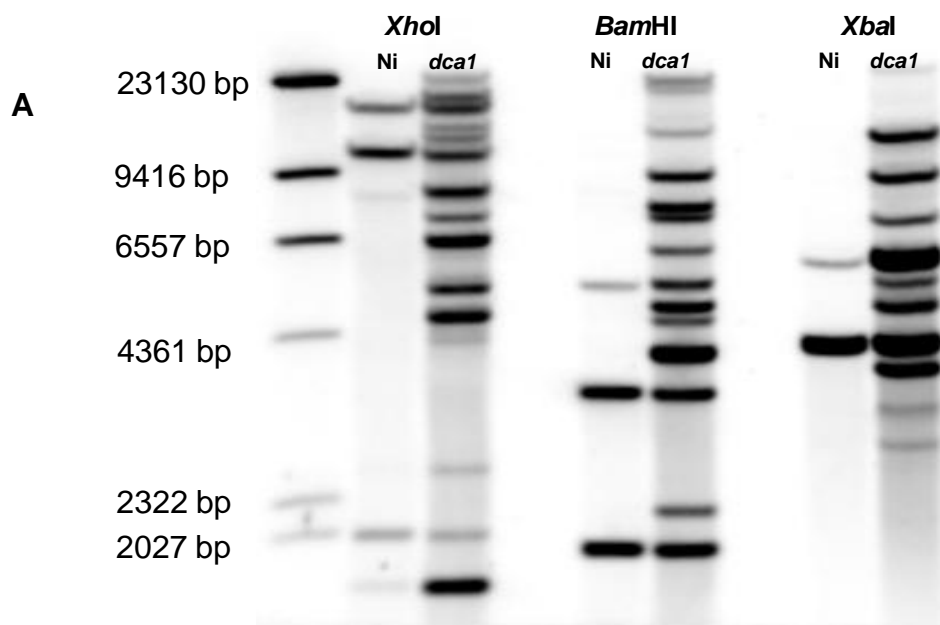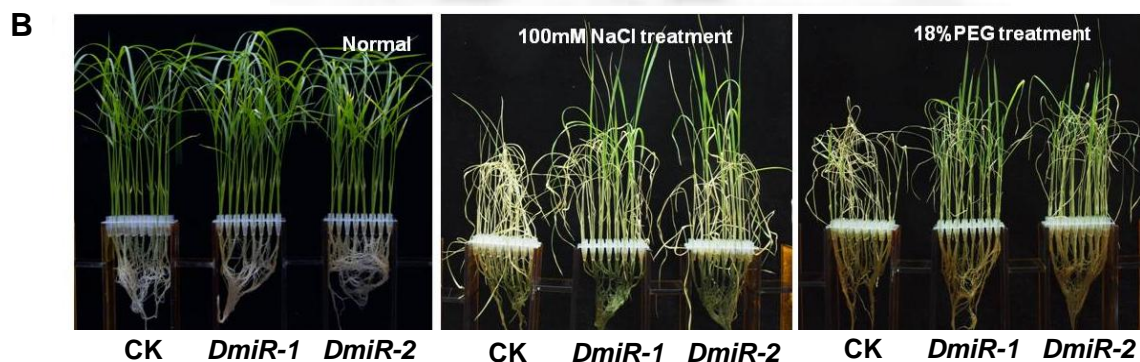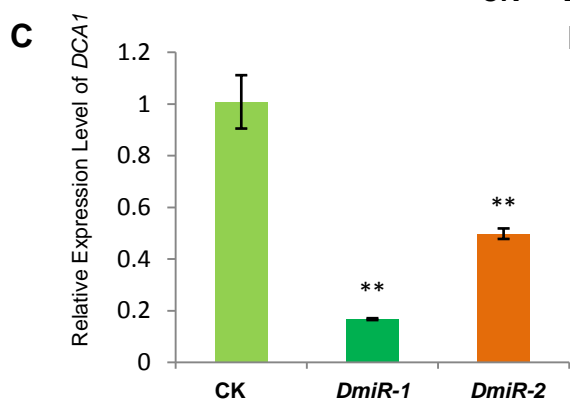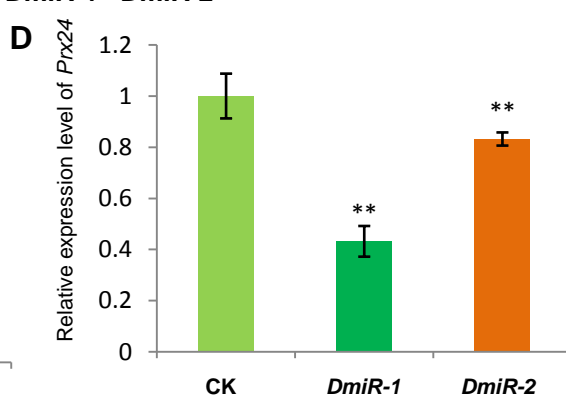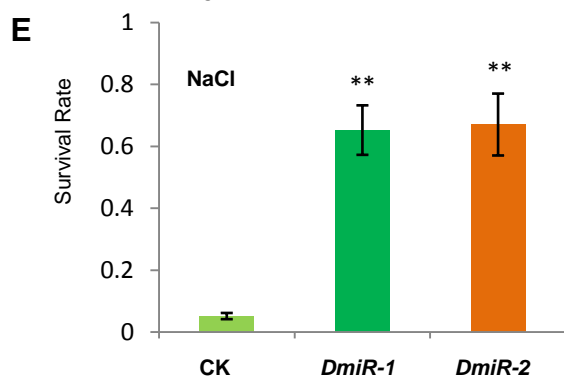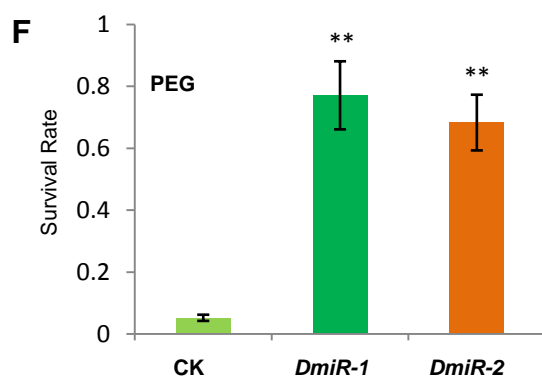

Supplement: S4 Fig — (A) Southern blot of Ni and dca1. (B) CK (vector in ZH11), DmiR-1 and DmiR-2 grown under normal conditions for 12 days (left), 12-day-old seedlings transferred to 100 mM NaCl for 11 days and recovered for 7 days (middle), or transferred to 18% PEG for 12 days and recovered for 7 days (right). (C) Expression levels of DCA1 normalized against actin. Data represent means ± sd (n = 3). (D) Expression levels of Prx24. Data represent means ± sd (n = 3). (E) Relative survival rates following NaCl treatment (n = 3 biological replicates, 24 plants of each replicate). Data represent means ± sd. (F) Relative survival rates following PEG treatment (n = 3 biological replicates, 24 plants of each replicate). Data represent means ± sd. Significant differences were determined using the Student’s t-test (**P <0.01). (PDF) [file pgen.1005617.s004.pdf]

**A**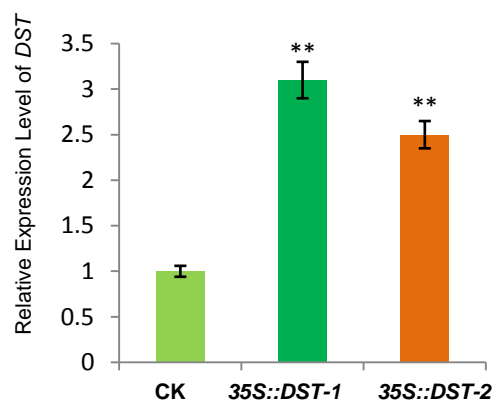**B**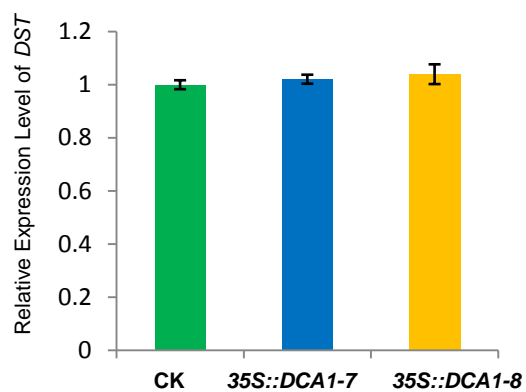**C**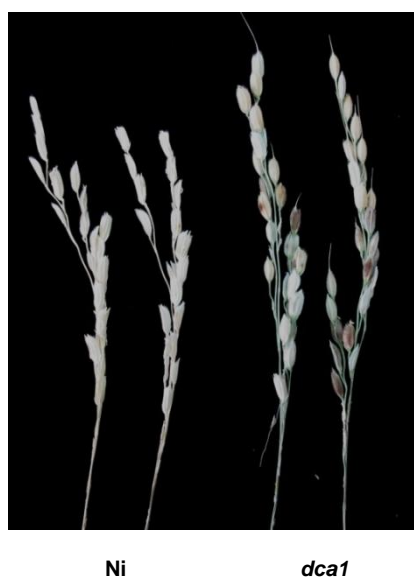**D**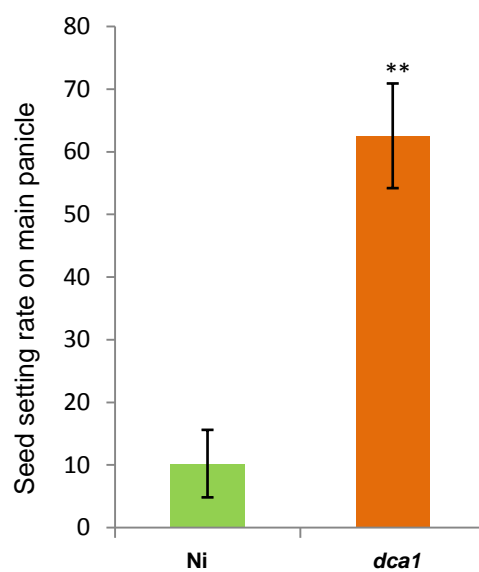

Supplement: S5 Fig — (A, B) Relative expression level of DST in 35S::DST and 35S::DCA1 plants grown under normal conditions. Samples were collected from 18-day-old seedlings. Data represent means ± sd (n = 3). (C, D) Seed setting on the main panicle of Ni and dca1 after soil dehydration treatment. Data represent means ± sd (n = 8). Significant differences were determined using the Student’s t-test (**P <0.01). (PDF) [file pgen.1005617.s005.pdf]

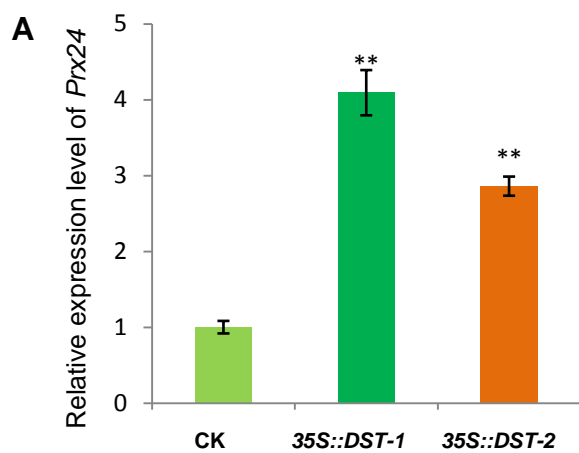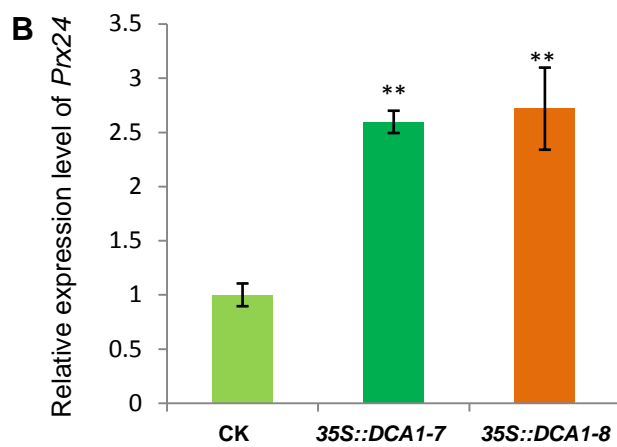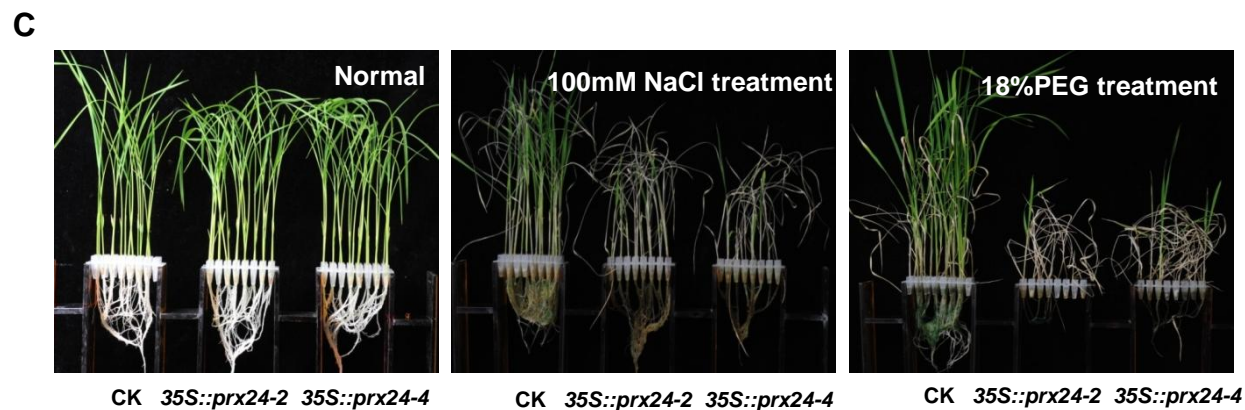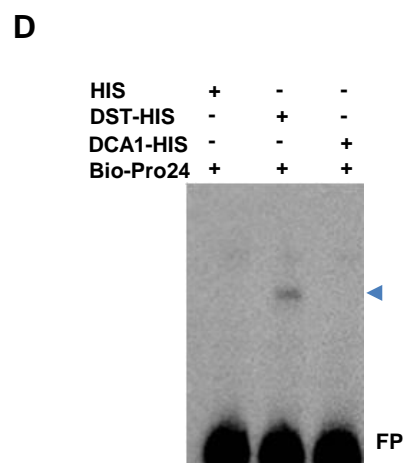

Supplement: S6 Fig — (A, B) Real-time PCR quantification of the DST downstream target gene Prx24 in CK, 35S::DST-1 and 35S::DST-2 (A) and CK, 35S::DCA1-7 and 35S::DCA1-8 (B). Data represent means ± sd (n = 3). (C) CK (vector in ZH11), 35S::Prx24-2 and 35S::Prx24-4 seedlings grown under normal conditions for 12 days (left), transferred to 100 mM NaCl for 9 days and recovered for 7 days (middle), or transferred to 18% PEG for 11 days and recovered for 7 days (right). (D) EMSA of DST and DCA1 proteins with biotin-tagged Pro24 (Bio-Pro24), a DST binding site-containing Prx24-promoter DNA sequence. The triangle indicates the shifted band. FP, free probe. Significant differences were determined using the Student’s t-test (**P <0.01). (PDF) [file pgen.1005617.s006.pdf]

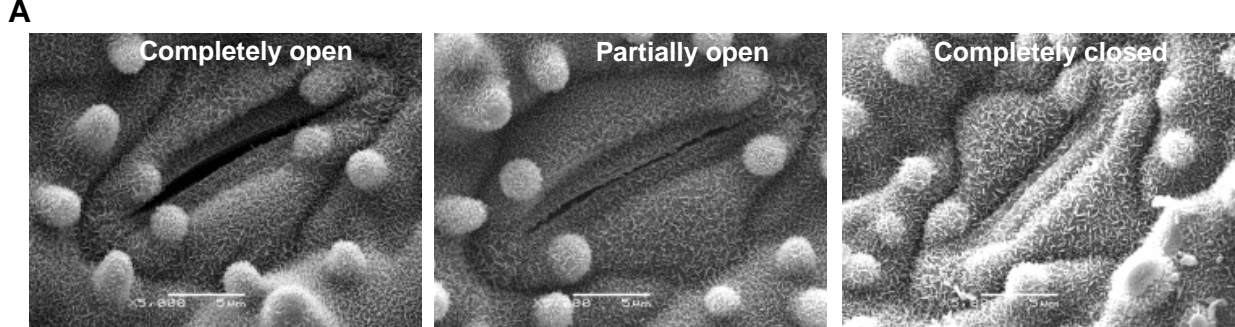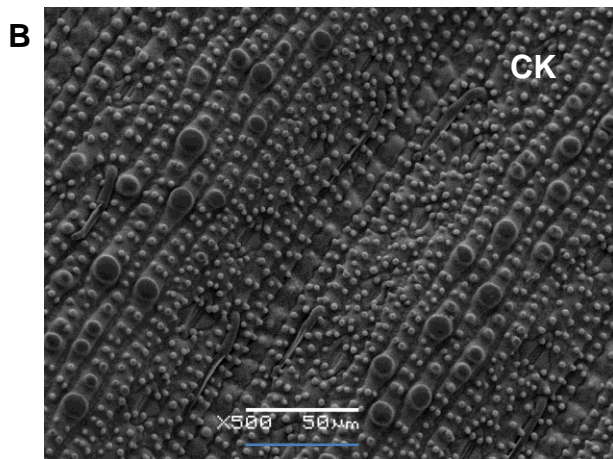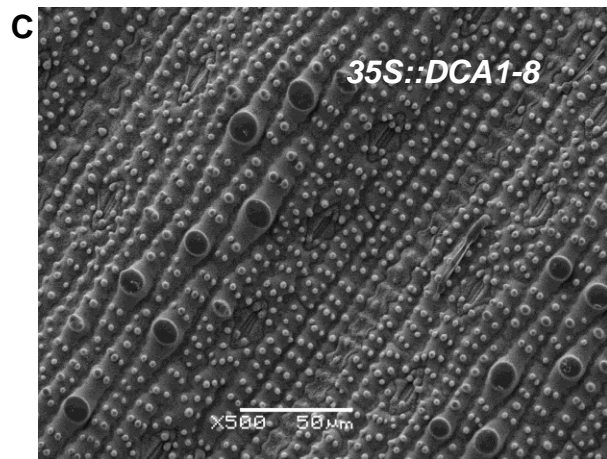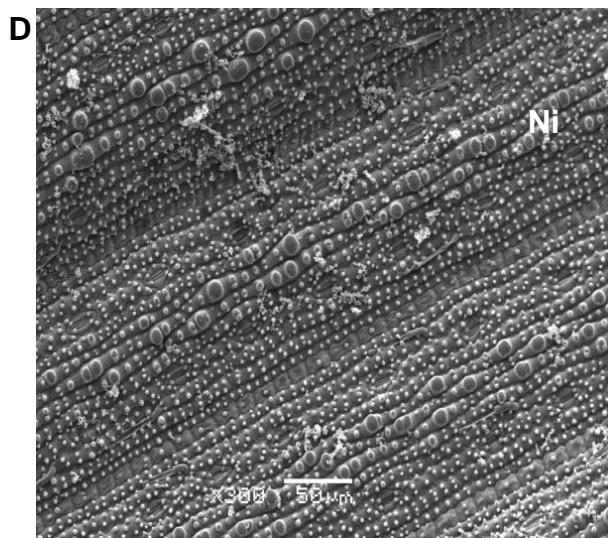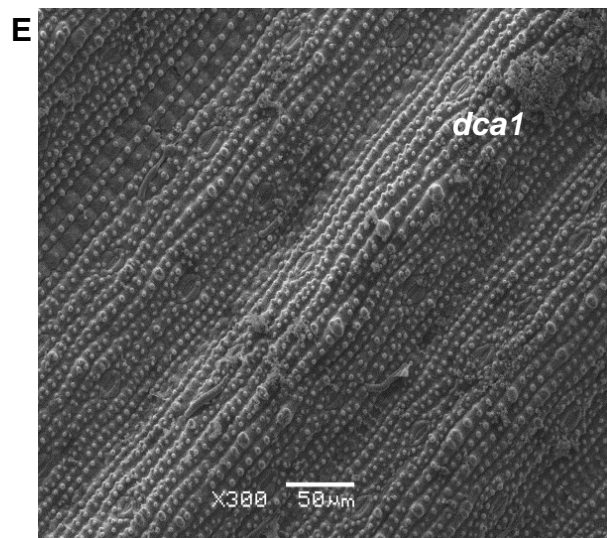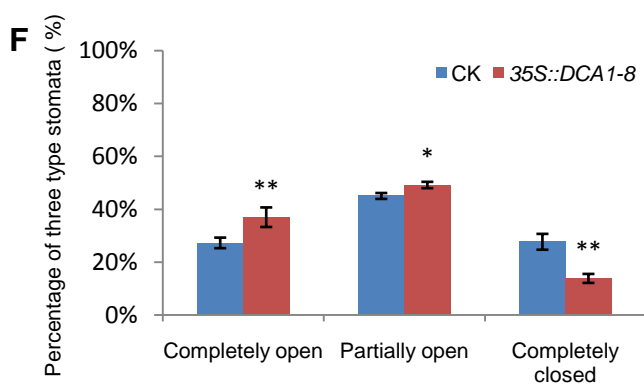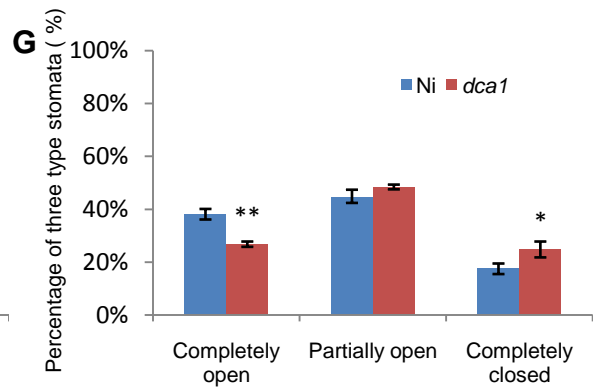

Supplement: S7 Fig — (A) Cryo scanning electron microscopy (Cryo SEM) images of three levels of stomatal opening of Ni. (B, C, D, E) Overview of stomatal status of CK (B), 35S::DCA1-8 (C), Ni (D) and dca1 (E) plants under low magnification. (F, G) Percentage of the three levels of stomatal opening in CK and 35S::DCA1-8 (F) and Ni and dca1 (G) under drought conditions (12% PEG for 6 h, n = 8 samples, 10 stomata per sample). Data represent means ± sd. Significant differences were determined using the Student’s t-test (*P<0.05, **P <0.01). (PDF) [file pgen.1005617.s007.pdf]

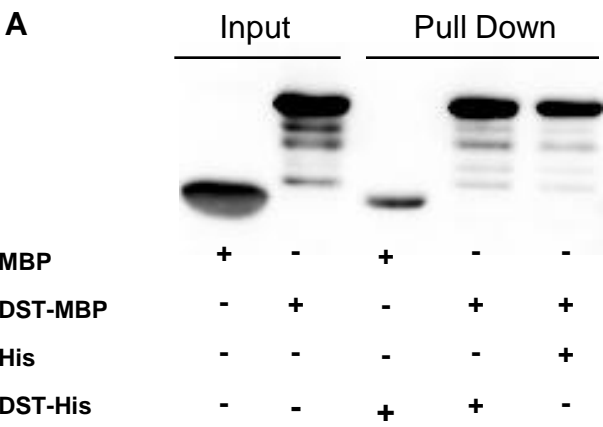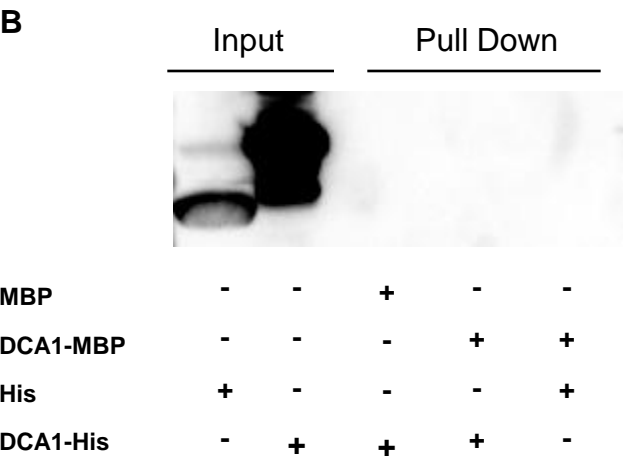

Supplement: S8 Fig — (A) In vitro pull-down of His2-tagged DST through MBP-tagged DST detected by immunoblotting with anti-MBP antibody. (B) In vitro pull-down of His2-tagged DCA1 through MBP-tagged DCA1 detected by immunoblotting with anti-His antibody. (PDF) [file pgen.1005617.s008.pdf]

**A**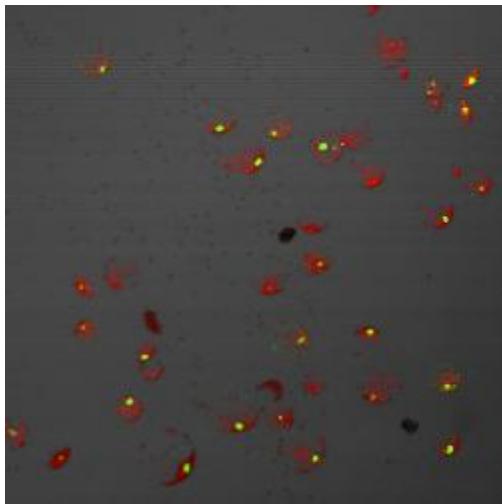**DST-DST BiFC 12h****B**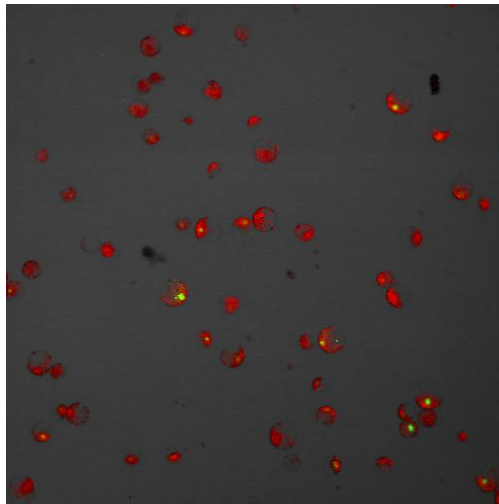**DST-DST BiFC 90mM NaCl 12h****C**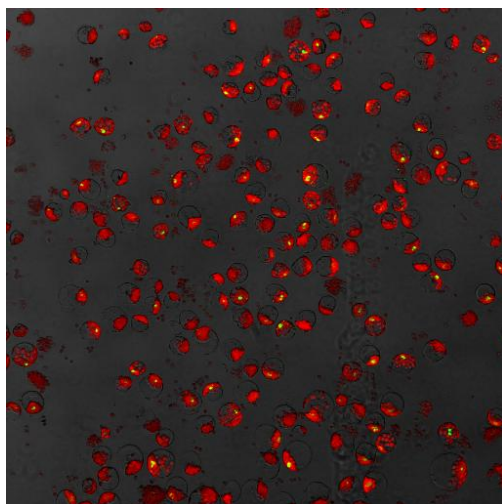**DST-DST BiFC 12h****D**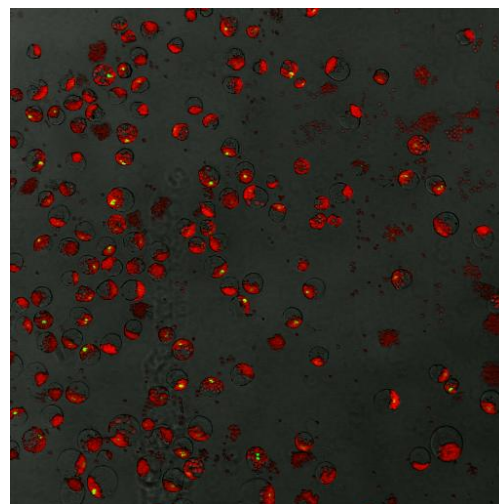**DST-DST BiFC 9h, 90mM NaCl & CHX 3h****E**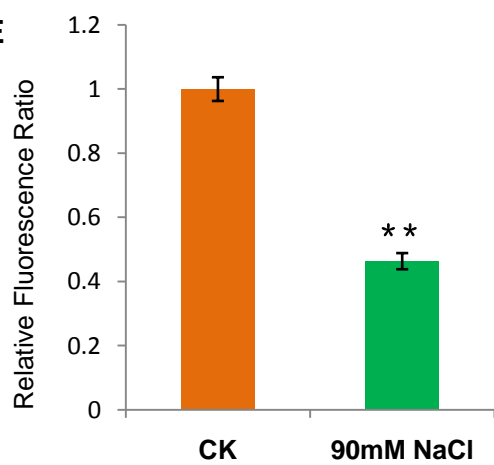**F**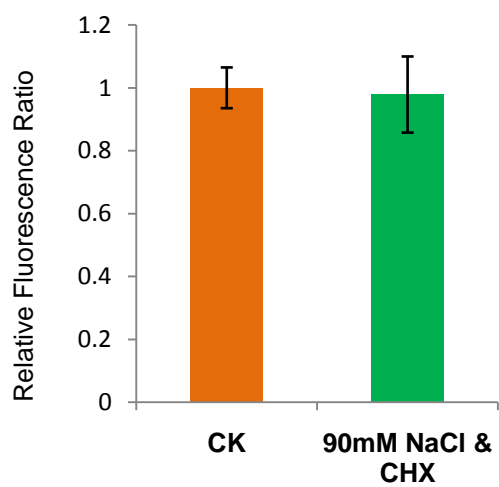

Supplement: S9 Fig — YFP fluorescence in the BiFC assay indicates dimerization of DST. (A, B) NaCl treatment reduces the dimeric form of DST (B) compared with normal conditions (A). (C, D) The dimeric form of DST is not influenced by NaCl treatment with cycloheximide (CHX) (D) compared with normal conditions (C). (E) Quantitative statistical analysis of (A) and (B) (n = 3 biological replicates, each containing 50 cells). Data represent means ± sd (n = 3). (F) Quantitative statistical analysis of (C) and (D) (n = 3 biological replicates, each containing 50 cells). Data represent means ± sd (n = 3). Significant differences were determined using the Student’s t-test (**P <0.01). (PDF) [file pgen.1005617.s009.pdf]
