# Supplementary material for: DCA1 Acts as a Transcriptional Co-activator of DST and Contributes to Drought and Salt Tolerance in Rice
Source: PLoS Genet. 2015 Oct 23;11(10):e1005617. doi: 10.1371/journal.pgen.1005617 (PMC4619773; doi:10.1371/journal.pgen.1005617)
Supplement: S1 Table — (PDF) [file pgen.1005617.s010.pdf]

S1 Table. Oligonucleotides used in this study.

Y2H	Sequence	Discription
△72	AAAGGATCCTGAAGGAGCGGAGCATCGGGTG	△72-pGBKT7
△72	AAAGTCGACCGAGGCTCAAGTTGAGGTCGAG	
DIP1BDF	catgCCATGGagatggaattggagtcggag	DIP1-pGBKT7
DIP1BDR	acgcGTCGACctcaaaccgtggagcgcgc	
DIP1ADF	ccATCGATagatggaattggagtcggag	DIP1-pGADT7
DIP1ADR	acgcGAGCTCctcaaaccgtggagcgcgc	
DSTADF	gGAATTCatggactccccgtcgcctatg	DST-pGADT7
DSTADR	cgGGATCCgctagaggctcaagttgag	
**qRT**		
qDIP1F	AATGCGGGTGTTGCTACAGT	DIP1
qDIP1R	ATGCGCTCTCATCACGTTCA	
qActF	TGGTCGTACCACAGGTATTGTGTT	Actin
qActR	AAGGTCGAGACGAAGGATAGCAT	
qPrx24F	AACCCATCCTACGCCAGA	Prx24
qPrx24R	CGCCTTGAGGTTCACGAAGTA	
qDSTF	CCAAGCTCCACCTACTCCTCTTCCAA	DST
qDSTR	GGGGGATTAATCACACACGAGGAGG	
**Mutant test**		
F	ATTGTTAGGTTGCAAGTTAGTTAAGA	
R	cagaattcgctcgagatccatctc	
**Dual luciferase assay**		
DIP1pGrnF	ggacccAAGCTTatggaattggagtcggagc	DIP1-pGREEN-64SK
DIP1pGrnR	acgcGTCGACtcaaaccgtggagcgcgcg	
DSTDBF	GGGaatggactccccgtcgcc	DST-pGAL4BD
DSTDBR	acgcGTCGACctagaggctcaagttgaggtcgag	
**BiFC**		
DIP1X6F	gcTCTAGAatggaattggagtcggag	DIP1-cYFP
DIP1X6R	acgcGTCGACtcaaaccgtggagcgcgcggctg	
DSTXY4F	gcTCTAGAatggactccccgtcgcc	DST-nYFP
DSTXY4R	acgcGTCGACgaggctcaagttgaggtcgag	
DSTXY6F	GGATCCatggactccccgtcgcc	DST-cYFP
DSTXY6R	GTCGACctagaggctcaagttgaggtcgag	
**Subcelluar location**		
DIP1YF	GCTCTAGAatggaattggagtcggagc	DIP1-YFP
DIP1YR	ggGGTACCaaccgtggagcgcgcggctg	
DSTYF	gcTCTAGAatggactccccgtcgcctatg	DST-YFP
DSTYR	acgcGTCGACgaggctcaagttgaggtcg	
**Protein expression**		
DSTcoldF	cgGGATCCatggactccccgtcgcctatg	DST-pCold TF
DSTcoldR	tgcTCTAGActagaggctcaagttgaggtcg	
DIP1coldF	ggacccAAGCTTatggaattggagtcggagc	DIP1-pCold TF

DIP1coldR	gcTCTAGAtcaaaccgtggagcgcgcg	
DSTMalF	GTCGACatggactccccgtcgcc	DST-pMAL-c5x
DSTMalR	GAATTCctagaggctcaagttgag	
DIP1MalF	catgCCATGGgcatggaattggagtcggag	DIP1-pMAL-c5x
DIP1MalR	agagGATATCaaccgtggagcgcgcggctg	
DSTHisF	AAAGGATCCTGATGGACTCCCCGTCGCCT	DST-pET32a
DSTHisR	AAAGTCGACCGAGGCTCAAGTTGAGGTCGAG	
**Gene Modifiation**		
DmiRI	AGTATGATGCGCTCTTATCGCGTCAGGAGATTCAGTTTGA	DmiR
DmiRII	TGACGCGATAAGAGCGCATCATACTGCTGCTGCTACAGCC	
DmiRIII	CTACGTGATGAGAGCGCATCATCTTCCTGCTGCTAGGCTG	
DmiRIV	AAGATGATGCGCTCTCATCACGTAGAGAGGCAAAAGTGA	
G-11491	cgGGATCCCAGCAGCAGCCACAGCAAA	
G-11494	gcTCTAGAGCTGCTGATGCTGATGCCAT	
DSTOEF	cccAAGCTTatggactccccgtcgcctatg	DST-OE
DSTOER	gcTCTAGActagaggctcaagttgaggtc	
DCA1OEF	ggacccAAGCTTatggaattggagtcggagc	DCA1-OE
DCA1OER	gcTCTAGAtcaaaccgtggagcgcgcg	
Prx24OEF	cgGGATCCatgcagggaggcgccaata	Prx24-OE
Prx24OER	gcTCTAGActaGGAGTTGACGGCGTTGC	
